# Systematic Analysis of the Maize *OSCA* Genes Revealing *ZmOSCA* Family Members Involved in Osmotic Stress and *ZmOSCA2.4* Confers Enhanced Drought Tolerance in Transgenic *Arabidopsis*

**DOI:** 10.3390/ijms21010351

**Published:** 2020-01-05

**Authors:** Liru Cao, Pengyu Zhang, Xiaomin Lu, Guorui Wang, Zhenhua Wang, Qianjin Zhang, Xin Zhang, Xin Wei, Fujian Mei, Li Wei, Tongchao Wang

**Affiliations:** 1 National Key Laboratory of Wheat and Maize Crop Science, College of Agronomy, Henan Agricultural University, Zhengzhou 450002, China; caoliru008@126.com (L.C.); zpyxiyuan@163.com (P.Z.); guorui9264@163.com (G.W.); meifujian0405@163.com (F.M.); 2 Grain Crops Research Institute, Henan Academy of Agricultural Sciences, Zhengzhou 450002, China; luxiaomin2004@163.com (X.L.); wzh201@126.com (Z.W.); zqjin@126.com (Q.Z.); zh5733764@126.com (X.Z.); weixin04@163.com (X.W.); 3 National Engineering Research Centre for Wheat, Zhengzhou 450002, China

**Keywords:** OSCAs, co-expression modules, proline content, DUF221 domain, abiotic stresses, interaction, drought resistance

## Abstract

OSCAs are hyperosmolality-gated calcium-permeable channel proteins. In this study, two co-expression modules, which are strongly associated with maize proline content, were screened by weighted correlation network analysis, including three ZmOSCA family members. Phylogenetic and protein domain analyses revealed that 12 ZmOSCA members were classified into four classes, which all contained DUF221 domain. The promoter region contained multiple core elements responsive to abiotic stresses and hormones. Colinear analysis revealed that *ZmOSCAs* had diversified prior to maize divergence. Most *ZmOSCAs* responded positively to ABA, PEG, and NaCl treatments. *ZmOSCA2.3* and *ZmOSCA2.4* were up-regulated by more than 200-fold under the three stresses, and showed significant positive correlations with proline content. Yeast two-hybrid and bimolecular fluorescence complementation indicated that ZmOSCA2.3 and ZmOSCA2.4 proteins interacted with ZmEREB198. Over-expression of *ZmOSCA2.4* in *Arabidopsis* remarkably improved drought resistance. Moreover, over-expression of *ZmOSCA2.4* enhanced the expression of drought tolerance-associated genes and reduced the expression of senescence-associated genes. We also found that perhaps *ZmOSCA2.4* was regulated by miR5054.The results provide a high-quality molecular resource for selecting resistant breeding, and lay a foundation for elucidating regulatory mechanism of *ZmOSCA* under abiotic stresses.

## 1. Introduction

Studies have shown that salt stress can cause osmotic stress and ionic stress on plants, and when stress is severe, it will cause extravasation of plant tissue, resulting in physiological drought [1]. Maize is sensitive to water, salt, and drought stress, which have become important factors limiting maize growth and yield. Studies have shown that in drought stress, maize in the big bell stage, the average silking period, and the filling period can lead to a reduction of 3%, 13%, and 4%, respectively [2]. For plants, high osmotic stress caused by salt and drought stress is the key environmental stress factor affecting maize growth and yield. The plants are exposed to external osmotic stress, and sudden changes in the concentration of solute around the cell. They broke the plants’ osmotic potential, and caused damage to their cell membrane. Osmotic regulation is an important physiological mechanism for plants to withstand water. Plants open the membrane channel by osmotic adjustment, and the permeate extracts water from the cells, thereby restoring normal cell volume [3,4]. These permeates include betaine, glycine, proline, etc. [5,6].

In eukaryotes, calcium is one of the main regulators of osmotic stress. Under osmotic pressure, intracellular calcium levels increase. This change can be achieved and regulated by calcium transport systems such as calcium channels and calcium pumps [7]. Plants are stimulated by the outside world to induce a rapid increase in intracellular calcium ion concentration, mainly by reacting themselves at physiological, cellular, and molecular levels, thereby inducing the expression of many stress-related genes and regulating plant tolerance to stress [8]. Ion channels are a class of membrane-integrated proteins that mediate ion transport and play a vital role in maintaining biological survival [9]. Analysis of the functional domain of the protein sequence revealed that the hyperosmolality-gated calcium-permeable channels (OSCA) family contained the Calcium-dependent channel (DUF221) domain [10]. Early detection of such proteins is also known as early responsive to dehydration stress protein (ERD4) [11] and trans-membrane channel-like proteins (TMC) [6]. Studies have shown that the transient increase of intracellular calcium concentration under drought stress is caused by the extracellular calcium influx induced by AtCSC1.2 protein under stress [7]. The predecessors systematically analyzed and identified the *OSCA* family of *Arabidopsis thaliana*, rice, and soybean, and found 15, 11, and 21 genes, respectively, located on the plasma membrane [12,13,14]. A maize gene *ZmERD4* was cloned and found that it can improve the drought resistance of *Arabidopsis* [15]. A drought-tolerant gene (*TaOSCA1.4*) was cloned in wheat, which belongs to the same gene family as *Arabidopsis AtOSCA1.8* and rice *OsOSCA1.4* and is related to grain number per ear and yield [16]. According to protein similarity, Ding divided 12 *OSCA* genes on maize into four subfamilies, and the transcriptional level and genetic variation found that *ZmOSCA4.1* was positively induced by drought stress [17]. It can be seen that plants can initiate genes involved in signal transduction and regulation of metabolic pathways to reduce the damage caused by drought or salt tolerance. Therefore, excavating and studying the potential salt and drought resistance gene and protein interaction network of maize is an important path for breeding highly resistant maize varieties.

To date, 12 *OSCA* genes have been identified from the protein sequence and gene structure in maize. Studies on the role of OSCA family in osmotic stress caused by salt, ABA, and drought exposure are limited, and the network regulatory mechanism and drought resistance involving OSCAs in maize remain unknown. In this study, based on transcriptome data from drought stress and rewatering, two co-expression modules, which are strongly associated with maize proline content, were screened by weighted correlation network analysis (WGCNA), including three ZmOSCA family members. Phylogenetic and protein domain analyses revealed that 12 ZmOSCA members were classified into four classes, which all contained DUF221 domain. The promoter region contained multiple core elements responsive to abiotic stresses and hormones. Colinear analysis revealed that *ZmOSCAs* had diversified prior to maize divergence. Most *ZmOSCAs* were positively induced by ABA, PEG, and NaCl treatments. *ZmOSCA2.3* and *ZmOSCA2.4* were up-regulated by more than 200-fold under the three stresses, and showed significant positive correlations with proline content. Yeast two-hybrid and bimolecular fluorescence complementation (BIFC) indicated that ZmOSCA2.3 and ZmOSCA2.4 proteins interacted with ZmEREB198.Over-expression of *ZmOSCA2.4* in *Arabidopsis* remarkably enhanced drought resistance. Moreover, over-expression of *ZmOSCA2.4* improved the expression of drought tolerance-associated genes (*MYB44*, *DREB2A,* and *NCED3*) and reduced the expression of senescence-associated genes (*SAG12*, *WRKY6,* and *BFN1*). We also found that perhaps *ZmOSCA2.4* was regulated by miR5054. The present analyses provide novel molecular resources for the breeding of new drought-tolerant cultivars, and provide a reference for future analysis the mechanism of *OSCA* genes under abiotic stress.

## 2. Results

### 2.1. Transcriptome Co-Expression Network Module Construction and Association Analysis Module and Proline Content

**Figure 1:**
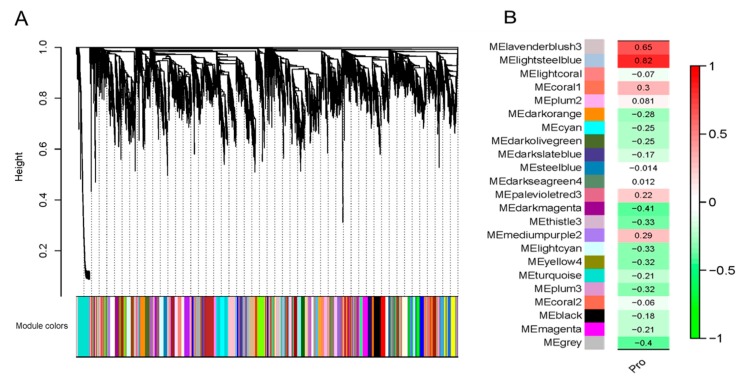
Weighted gene co-expression network analysis (WGCNA) gene expression module of drought stress and rewatering transcriptome sequencing and correlation analysis between module and proline content. (**A**) Each color represents a module. (**B**) The number represents the correlation coefficient between proline content and each module, and the green to red indicates the maximum negative correlation to the maximum positive correlation. Pro stands for proline content.

In this study, weighted gene co-expression network analysis (WGCNA) was used to analyze the genes represented among drought stress and rewatering transcriptome sequences. Co-expression network analysis showed that 8169 drought response genes could be clustered into 23 co-expression modules under drought stress (Figure 1A, Table S1). Correlation analysis was performed on these 23 co-expression modules and maize proline content, and two co-expression modules (MElavenderblush3 and MElightsteelblue) that significantly correlated with proline content were detected (Figure 1B). The proline is an important osmotic adjustment substance. Previous studies have shown that proline can maintain the intracellular osmotic balance, thereby reducing the severity of cell damage when plants exposed to osmotic stress [18,19]. Three genes expressed in the two modules contained DUF221 domain, which were characteristic of calcium-permeable stress-gated channel proteins (Table S1). Our research aims to characterize the maize OSCA family members, their responses to different modes of osmotic stress, and the network of mechanisms that control their expression and function.

### 2.2. Genome-Wide Identification and Classification of OSCA Genes in Maize

**Figure 2:**
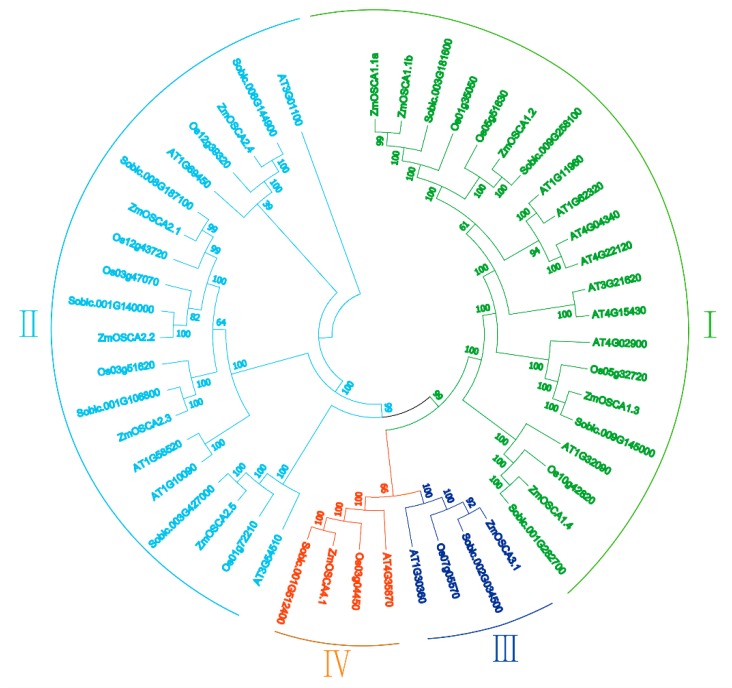
Complex phylogenetic tree of OSCAs in *Arabidopsis*, sorghum, rice, and maize. An unrooted tree is generated with the MEGA5.2 software using the amino acid sequences of the OSCA proteins by the neighbor-joining (NJ) method, with 1000 bootstrap replicates. The tree shows four major phylogenetic classes (I to IV) indicated with different colored backgrounds.

The maize genome (genome version: AGPv3.0) was searched by a Hidden Markov Model using DUF221 domain (Pfam login number: 02714). Finally, 12 genes in maize genome were found to be *ZmOSCAs*, named after *Arabidopsis* orthologs. The result was consistent with the Ding (2019) [17]. To investigate the phylogenetic relationships of OSCA proteins in maize, *Arabidopsis* (a dicotyledon model plant), and sorghum and rice (C4 monocotyledon model plants), we performed a phylogenetic analysis of 49 OSCA protein sequences. The OSCA proteins were resolved into four major classes: Classes I, II, III, and IV (Figure 2). Class I contained 21 OSCA members, belonged to the largest branch, followed by class II. Almost all subfamilies of OSCA contained orthologs, and more members had more orthologs. Compared 12 maize OSCAs with sorghum, *Arabidopsis,* and rice, there were more orthologs pairs in sorghum and rice. There was more homology with sorghum. *ZmOSCA1.1a* and *ZmOSCA1.1b* were also found to be repetitive events in the maize genome. The results showed that the OSCA family had experienced a similar evolutionary history in the maize, sorghum, and rice genomes, and maize OSCA family members showed a closer phylogenetic relationship with those of sorghum. In addition, a replication event in the maize genome resulted in duplication of one OSCA gene after phylogenetic divergence of maize, sorghum, and rice.

The *ZmOSCA* genes were randomly located on chromosomes 1, 3, 5, 6, and 8 of maize, and encoded polypeptides of 249 to 810 amino acids. Chromosomes 1 and 3 carried up to three *ZmOSCA* genes, and chromosomes 5 and 8 harbored two genes. By contrast, chromosomes 6 and 9 each carried only one *ZmOSCA* gene. The number of exons per *ZmOSCA* gene varied from one to 11. Two-thirds (8/12) of *ZmOSCA* genes contained more than eight exons, and only 17% (2/12) contained fewer than two exons. The predicted molecular weights of the ZmOSCA proteins ranged from 28.68 to 93.88 kDa, and the pI ranged from 7.67 to 9.35. Half of 12 ZmOSCAs contained more than nine transmembrane regions. (Table 1).

### 2.3. Additional Conserved Motifs in OSCA Genes in Maize

**Figure 3:**
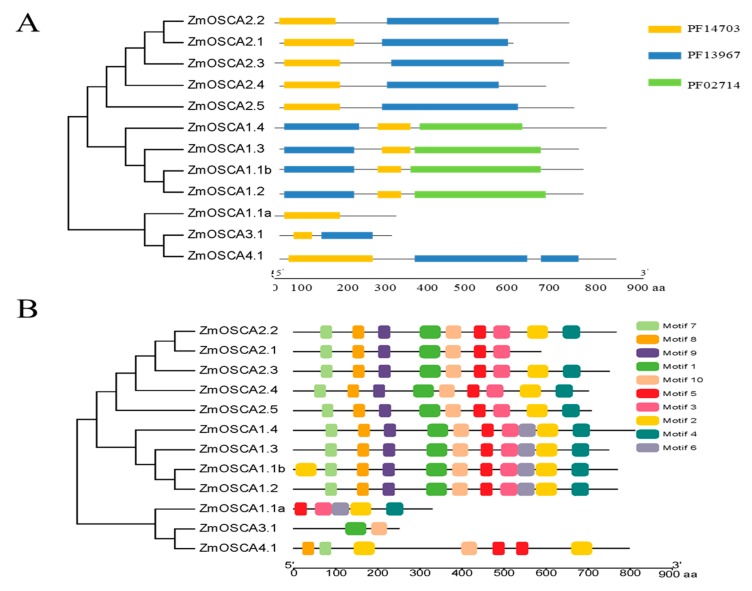
Distribution of functional domains and conserved motifs in ZmOSCA proteins. (**A**) The phytozome website queries the functional domain of the protein encoded by the *ZmOSCA* genes. PF14703, PF13967, and PF02714 represent the Cytosolic domain, late exocytosis, and Calcium-dependent channel domains, respectively. Gray lines represent amino acid sequences, and each rectangle length represents the amino acid length of the domain; (**B**) all motifs were identified by MEME using the complete amino acid sequences of ZmOSCA proteins. Different motifs are indicated by different colors boxes numbered 1–10, and the length of each box in the proteins does not represent the actual motif size. The annotation of each motif is listed on the right. The regular expression sequences of the motifs 1–10 are listed in Table S2.

Analysis of the protein domains of these genes revealed that ZmOSCA1.1a contained only Calcium-dependent channel (PF02714), ZmOSCA3.1 contained only Cytosolic domain (PF14703), and ZmOSCA4.1 contained Calcium-dependent channel (PF02714) and Cytosolic domain (PF14703) structure, but proteins of the other nine genes also contain late exocytosis (PF13967), as shown in Figure 3A. To further explore potential function of these proteins, we performed additional conserved motifs analysis using the MEME tool, which revealed 10 additional conserved motifs apart from the protein sequences (Figure 3B, Table S2). 

Motif 5 was present in all classes (except ZmOSCA3.1), whereas motif 3 was present in classes I and II. Motifs 5 and motifs 3 were located in the calcium-dependent channel domain, which was a basic conserved domain shared by members of the OSCA family. Motifs 7 and 8 were present in all genes except ZmOSCA1.1a and ZmOSCA3.1. Motifs 7 and 8 were located in the late exocytosis domain, which indicated that these two genes did not currently perform this function. The majority of conserved motifs were restricted to specific classes, which was indicative of functional differences among classes. Motif 6 was only detected in classes I, which indicated functional similarity among members within this classes (Figure 2). Classes-specific motifs can be useful for determining the specific function of each classes. We also detected multiple motifs within the same class. For example, ZmOSCA2.1 in classes II lacked motifs 2, 4, and 6, whereas the other four genes in the same classes contained the same motifs except motif 6. Thus, we speculated that these four genes (ZmOSCA2.2, -2.3, -2.4, and -2.5) shared similar functions. This phenomenon was also observed in other classes, and suggested that there were different action mechanisms within each classes. The results of the conserved motif analysis were generally consistent with the evolutionary relationships implied from the phylogenetic analysis.

### 2.4. Cis-Element Analysis of OSCA Gene Promoter Sequences

Genes contain many cis-acting elements in their promoter regions that participate in various pathways, for example, the ABA response signal transduction pathway and abiotic stress response pathway [20]. In addition to some basic core components, multiple cis elements were also found in the promoter regions, such as ABRE, ARE, DRE core, and MBS, LTR (Figure S1). These cis-elements are key components for abiotic stress responsiveness. Other *ZmOSCA* genes all contained ABRE response elements except *ZmOSCA4.1.* Classes I and II contained cis-elements associated with water deficiency, such as DER and MBS, but genes in classes III and IV did not. For the genes of classes II, only *ZmOSCA2.5* gene did not contain ARE element, but only *ZmOSCA2.5* and *ZmOSCA2.2* contained MBS element, and only *ZmOSCA2.4* contained LTR elements. The cis-elements of the *ZmOSCAs* differed within and among classes. Together, these results indicated that the genes in the same class may have different mechanisms of action, and genes of different classes may work together. 

### 2.5. Collinearity Relationships of OSCA Genes among Maize, Sorghum, Rice, and Arabidopsis

**Figure 4:**
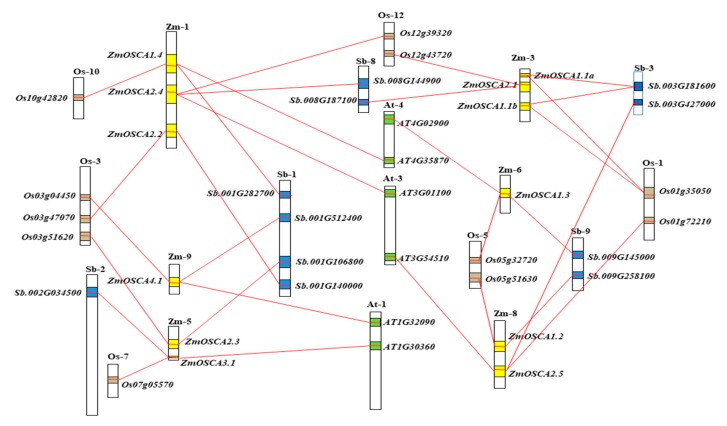
Collinearity relationships of *OSCA* genes among Maize, Sorghum, rice, and *Arabidopsis*. Zm, Sb, Os, and At stand for chromosomes in maize, sorghum, rice, and Arabidopsis, respectively. Each pair of *OSCA* homologous genes is connected by a red line. The yellow, blue, brown, and green boxes represent homologous regions in the genomes of maize, sorghum, rice, and *Arabidopsis*.

Comparative genomics analysis to detect gene collinearity indicates homologous gene function and interspecific phylogenetic relationships. Researches on the collinearity of *OSCA* genes showed that *ZmOSCA1.4*, *ZmOSCA2.4*, *-4.1*, *-3.1*, *-1.3,* and *ZmOSCA2.5* were colinear with sorghum, rice, and *Arabidopsis*, and the other six *ZmOSCA* genes were only colinear with sorghum and rice (Figure 4). It suggested that large-scale expansion probably did not occur before the monocot-dicot split, but large-scale expansion occurred before maize-sorghum and maize-rice division. Combined with the tight phylogenetic relationship between ZmOSCA proteins and SbOSCA proteins, it was further speculated that large-scale expansion occurred before maize-sorghum split. In addition, both *ZmOSCA1.1a* and *ZmOSCA1.1b* had a collinear relationship with the *sb.oo3g181600* and *Os01g35050* genes. These results were consistent with those of the phylogenetic analysis (Figure 2), which also confirmed the accuracy of our analysis. Combining the paralogs *ZmOSCA1.1a* and *ZmOSCA1.1b*, it can be further stated that, although the majority of *ZmOSCA* genes existed before divergence of maize, certain *ZmOSCA* genes may have originated as a result of maize genome duplication.

### 2.6. Expression of ZmOSCA Genes under Abiotic Stress

**Figure 5:**
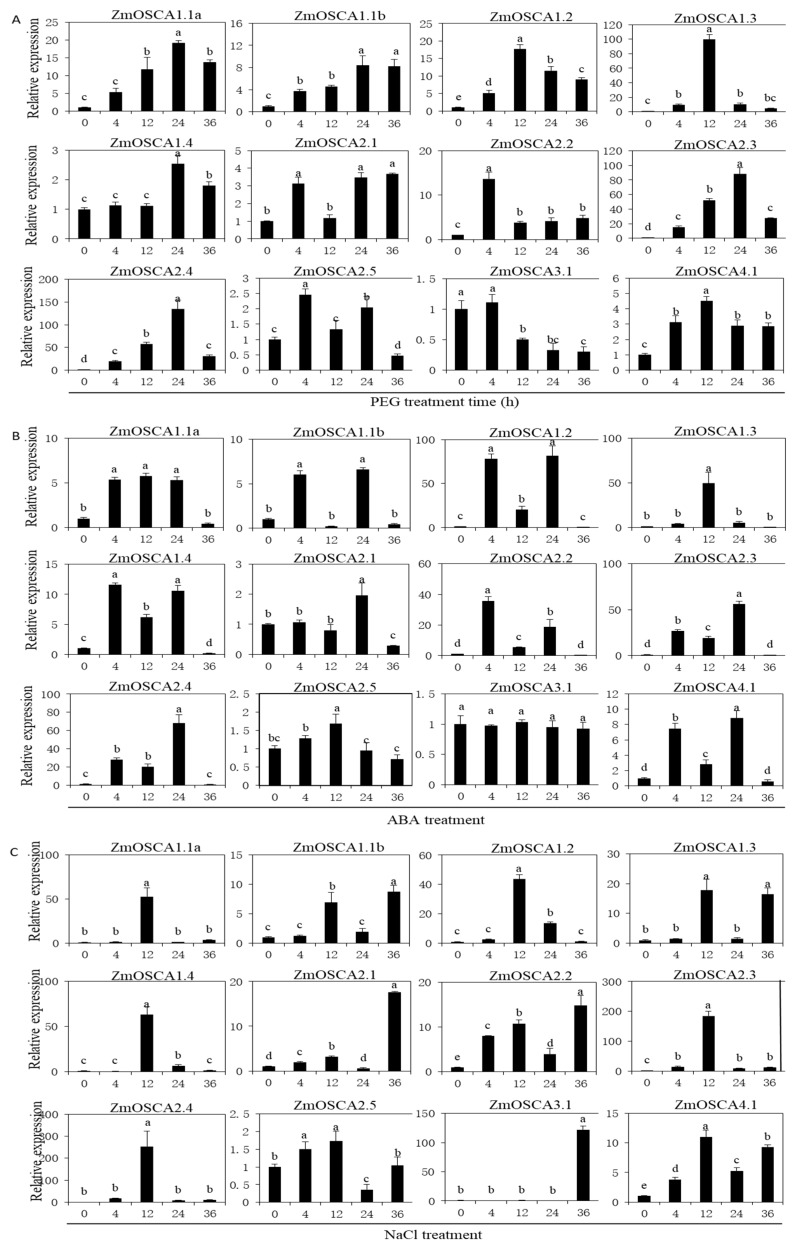
Expression patterns of *ZmOSCA* genes in response to PEG, ABA, and NaCl treatments. The relative expression level of 12 *ZmOSCA* genes was examined by the qRT-PCR. (**A**) Relative expression of 12 *ZmOSCA* genes under PEG treatment at 0, 4, 12, 24, and 36 h; (**B**) relative expression of 12 *ZmOSCA* genes under NaCl treatment at 0, 4, 12, 24, and 36 h; (**C**) relative expression of 12 *ZmOSCA* genes under ABA treatment at 0, 4, 12, 24, and 36 h; the error bars represent standard deviations (SD); t-test was *p* < 0.05, and the different letters represented a significant difference in the relative expression between samples. The y-axes are scales of relative expression level and x-axes are the time course of treatments for each condition.

Previous studies have shown that plants respond to and adapt to drought and high salt stress by inducing a range of gene expression [21]. PEG and NaCl stresses are often associated with each other and may cause similar cellular damage [22], because osmotic stress is the first and major component of drought and salt stresses when plants are exposed to high NaCl concentrations and water-deficient environments [23]. ABA is an important plant stress signaling hormone that can be synthesized in response to various abiotic stresses and regulates the expression of numerous stress-responsive genes in plants [24]. Analysis of expression patterns revealed that with the exception of *ZmOSCA3.1*, 11 other genes were upregulated by PEG, NaCl, and ABA treatments. *ZmOSCA3.1* was significantly down-regulated at 12 h under PEG stress, whereas its expression did not change under ABA treatment and was significantly up-regulated at 36 h under NaCl stress. (Figure 5). Further analysis showed that, in the case of PEG stress, the expression of *ZmOSCA2.3* and *ZmOSCA2.4* increased by a factor of 10 to 100-fold compared with CK (0h). Under ABA treatment, the expression level of genes increased by a factor of 10 to 100-fold compared with CK except *ZmOSCA2.1*, *ZmOSCA2.5,* and *ZmOSCA3.1*. Under NaCl stress, the expression level of genes increased by a factor of 10 to 100-fold compared with CK except *ZmOSCA2.5*. *ZmOSCA1.2*, *-1.3*, *-2.3,* and *-2.4* were expressed simultaneously in response to ABA, PEG, and NaCl treatments. Under the different types of osmotic stress, gene response patterns showed similarities and differences. These results indicated that maize *OSCAs* strongly responded to osmotic stress caused by PEG, NaCl, and ABA treatment, and provided additional clues for investigation of the physiological function of *ZmOSCAs* as osmosensors in maize.

### 2.7. Analysis of the Relationship between ZmOSCAs Gene and Proline Content

**Figure 6:**
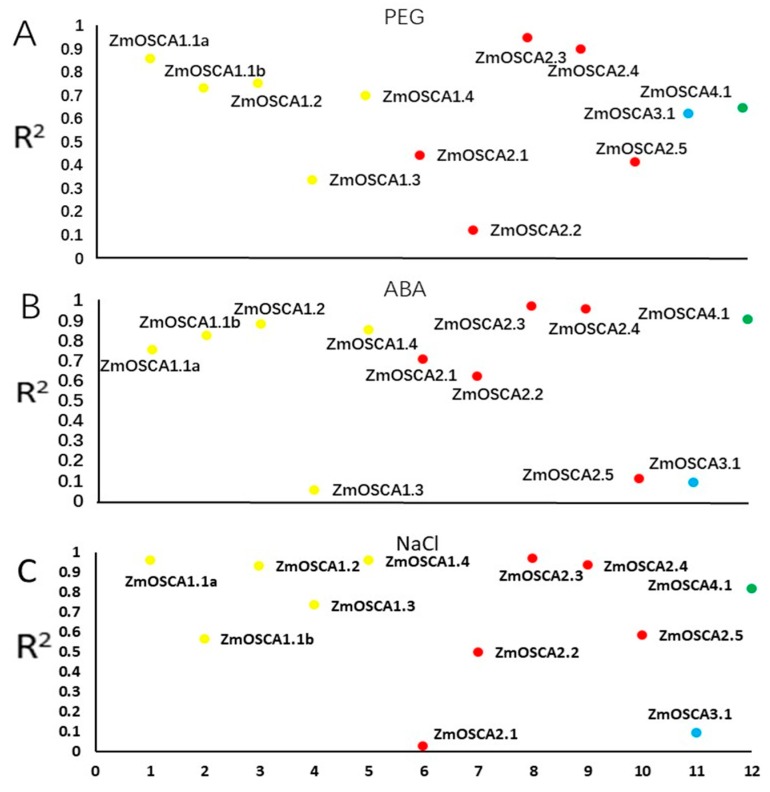
The relationship between *ZmOSCAs* gene and proline content. (**A**) Correlation between gene expression and proline content under PEG treatment. (**B**) Correlation between gene expression and proline content under ABA treatment. (**C**) Correlation between gene expression and proline content under NaCl treatment. The yellow, red, blue, and green circles represent the class I, II, III, and IV members, respectively. The y-axis is the absolute value of the coefficient of correlation between gene expression and proline content, and the x-axis represents the number of genes.

As a key osmotic adjustment substance, proline reduces the osmotic potential by increasing the accumulation amount under salt, drought, and other abiotic stresses, maintaining cell stability and alleviating the damage caused by stress [25]. The correlation between the expression level of each *ZmOSCA* gene and the proline content in leaves at the corresponding time points under the three osmotic stresses was analyzed. The correlation coefficients between the expression level of the majority of genes and proline content under the three stresses were higher than 0.56. (Figure 6 and Table S3). The correlation coefficients for *ZmOSCA2.3*, *-2.4*, and *-1.1a, ZmOSCA1.1b*, *-2.3*, *-2.4*, *-4.1*, *-1.2*, and *-1.4,* and *ZmOSCA2.3*, *-1.4*, *-1.1a*, *-2.4*, *-1.2*, and *-4.1* were higher than 0.82, respectively, under treatment of PEG, ABA, and *NaCl.ZmOSCA3.1* was negatively correlated with proline content (-0.618) under PEG treatment, but no significant correlation was observed under the ABA and NaCl treatments. Thus, differences in the mechanism of action of *ZmOSCA* genes in response to the different abiotic stresses were observed. Among these genes, the expression levels of *ZmOSCA 2.3* and *ZmOSCA 2.4* were highly correlated with proline content under all three treatments. In general, genes that were highly correlated with proline content may play an important role in response to osmotic stress, but the degree of response of each gene was indicated to differ.

### 2.8. Predicting the Protein Interaction Network of ZmOSCAs

Systematic analysis of the interaction of ZmOSCA family proteins in biological systems is important to understand the reaction mechanisms of signal and energy metabolism under abiotic stress and to understand the functional linkages among proteins. Therefore, we explored the possible network of regulatory mechanisms of the ZmOSCAs. 

The 12 ZmOSCA interaction proteins were divided into six interaction clusters, such as ZmOSCA1.1a, ZmOSCA1.1b, ZmOSCA1.2, and ZmOSCA3.1. Similar results were obtained for ZmOSCA2.1 and ZmOSCA2.2. However, ZmOSCA1.3, ZmOSCA1.4, and ZmOSCA4.1 each formed a unique protein interaction cluster (Figure S2). Interestingly, ZmOSCA2.3, ZmOSCA2.4, and ZmOSCA2.5 in class II were predicted to interact with transcription factors AP2/EREBP, EREB195, EREB134, ZmEREB198, bHLH35, and bHLH31 and functional genes PSGL-1b, QPRT, and Gpi7 (Figure 6), which may show a similar mechanism of action. ZmOSCA proteins in different classes and in the same class also showed the same interaction protein. For example, ZmOSCA1.1a, ZmOSCA1.1b, ZmOSCA1.2, and ZmOSCA3.1 were both predicted to interact with 6PGL4, MIS12-1, RAD51C, PER70, HIS4, CASP-4, and MtN21a (Figure S2). These proteins were predominantly involved in plant metabolism, sugar synthesis, the antioxidant system, as well as other processes (Table S4), indicating that proteins in different and same groups jointly responded to plant stress through synergistic action.

### 2.9. Yeast Two-Hybrid and Bimolecular Fluorescence Complementation Validate the Interaction Proteins of ZmOSCA2.3 and ZmOSCA2.4

**Figure 7:**
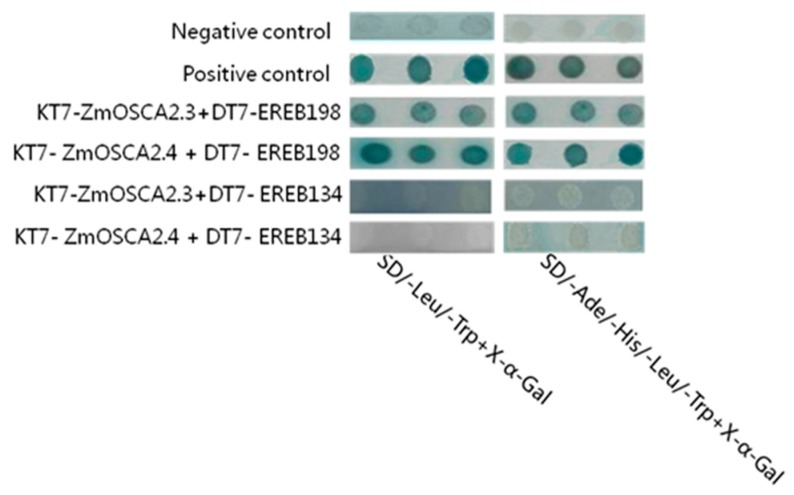
The yeast two-hybrid analysis for interactions between ZmOSCA2.3, ZmOSCA2.4, and ZmEREB198, EREB134 proteins. Negative control (pGADT7-T + pGBKT7-Lam); positive control (pGADT7-T + pGBKT7-53); KT7 and DT7 represent pGBKT7 and pGADT7 vectors, respectively. The blue spots in the figure represent an interaction between the two proteins.

The expression levels of *ZmOSCA2.3* and *ZmOSCA2.4* were significantly increased under three stresses (Figure 5), and expression levels of the two genes were significantly positively correlated with proline content under each stress (Figure 6). These results indicated that the two genes play an important role in the plant response to osmotic stress. On the basis of the predicted interaction proteins, we verified the interaction of ZmOSCA2.3 and ZmOSCA2.4 with transcription factors using yeast two-hybrid in vitro and BIFC in vivo assays. The yeast two-hybrid assay showed that the ZmOSCA2.3 and ZmOSCA2.4 proteins interacted with the transcription factor ZmEREB198, but did not interact with EREB134 (Figure 7). The BIFC produced identical results (Figure S3). These results also suggested that a certain degree of false positives may be associated with STRING predictions of protein interactions.

### 2.10. Over-Expression of ZmOSCA2.4 in Arabidopsis Enhances Plant Tolerance to Drought Stress

**Figure 8:**
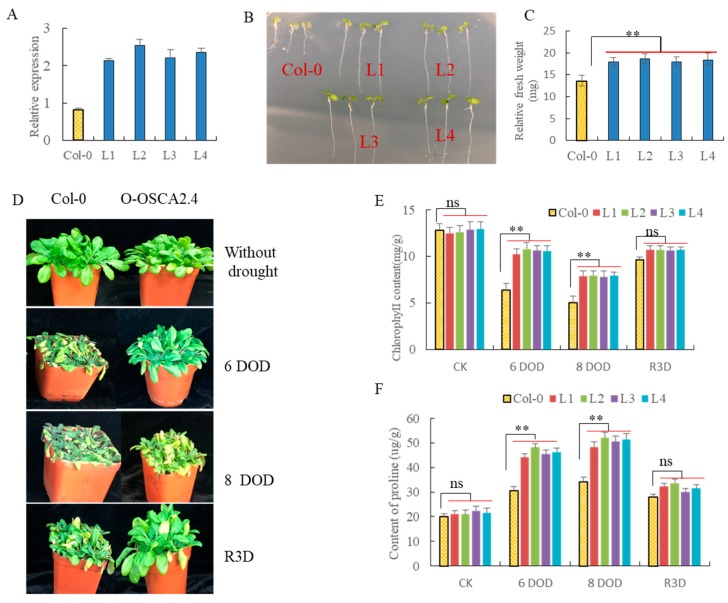
Over-expression of *ZmOSCA2.4* in *Arabidopsis* enhances plant tolerance to drought stress. (**A**) Quantitative RT-PCR analysis of *ZmOSCA2.4* expression in transgenic *Arabidopsis*.Col-0 is wild type, and L1–L4 are *ZmOSCA2.4* transgenic lines; (**B**) one-week-old seedlings were transferred and grew for 3 days on MS medium supplemented with 300 mM Mannitol; (**C**) the relative fresh weight of *ZmOSCA2.4* transgenic *Arabidopsis* and Col-0 under 300 mM Mannitol. Fresh weight in the picture represents three plants of each material; (**D**) O-OSCA represents *ZmOSCA2.4* transgenic *Arabidopsis* containing L1–L4. Col-0 and O-OSCA plants were exposed for a period of six to eight days of drought (DOD). The plants were then rewatered for three days (R3D) and photographed; (**E**) statistical analysis of chlorophyll content of leaves of wild-type (Col-0) and transgenic lines (O-OSCA2.4) treated with drought for six days, eight days, and rewatering for 3 days; (**F**) measurement of proline content in leaves of wild-type (Col-0) and transgenic lines (O-OSCA) treated with drought for six days, eight days, and rewatering for three days. Mean values and standard errors were shown from three independent experiments. T tests for equality of means demonstrated that there was very significant difference between wild type (Col-0) and transgenic lines (** *p* value < 0.01).

The coding sequence of *ZmOSCA2.4* fused to the CaMV 35S promoter were introduced into *Arabidopsis*, and four homozygous transgenic lines (T3 generations) were obtained. Expression levels of *ZmOSCA2.4* in the transgenic plants were examined by qRT-PCR analysis (Figure 8A). The four transgenic lines (L1–L4) with higher *ZmOSCA2.4* expression were selected for analyzing their phenotypes under drought stress. When one-week-old seedlings grew on MS medium under normal conditions, there was no difference between Col-0 and transgenic *Arabidopsis*. When one-week-old seedlings were transferred and vertically cultured for 3 days on MS medium containing 200 mM mannitol concentration (Figure 8B), the fresh weight of the transgenic lines was significantly greater than Col-0 (Figure 8C) and *ZmOSCA2.4* transgenic lines displayed better drought tolerance than Col-0.

The *ZmOSCA2.4* transgenic *Arabidopsis* were compared to Col-0 plants after a drought period of six to eight days and rewatering for three days. As shown in Figure 8D, *ZmOSCA2.4* transgenic lines have better drought resistance than Col-0. Statistical analysis indicated that there were significant differences in chlorophyll content, and proline content between *ZmOSCA2.4* transgenic lines and Col-0 under drought stress (Figure 8E,F). The over-expression of *ZmOSCA2.4* significantly enhanced the drought resistance of *Arabidopsis,* indicating that *ZmOSCA2.4* played an important role in drought resistance.

### 2.11. Analysis of Drought Tolerance-Associated and Senescence-Associated Genes Expression

**Figure 9:**
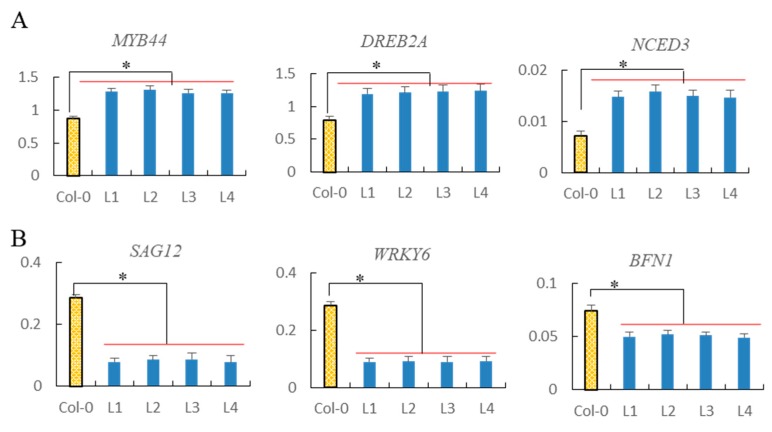
Analysis of drought tolerance-associated and senescence-associated genes expression. (**A**) Levels of *MYB44* (*At5g67300*), *DREB2A* (*At5g05410*) and *NCED3* (*At3g14440*) mRNA were determined relative to *ACTIN2* (*At3g18780*) using qRT-PCR. These genes are associated with drought stress; (**B**) levels of *SAG12* (*At5g45890*), *WRKY6* (*At1g62300*), and *BFN1* (*At1g11190*) mRNA were determined relative to *ACTIN2* (*At3g18780*) using qRT-PCR. Those are senescence-associated genes. Data represent at least three independent experiments using RNA extracted from leaves of Col-0 and *ZmOSCA2.4* transgenic *Arabidopsis* (L1–L4) subjected to eight days of drought. Statistically significant differences (* *p* < 0.05) are represented by an asterisk.

As shown in Figure 9, *ZmOSCA2.4* could improve the drought resistance of *Arabidopsis*. We also analyzed transcription of some genes involved in drought tolerance, such as *MYB44* (*At5g67300*), *DREB2A* (*At5g05410*), and *NCED3* (*At3g14440*). The mRNA levels of these three genes increased in *ZmOSCA2.4* lines after eight days of drought than Col-0 plants (Figure 9A).

To further investigate the senescence process, mRNA levels of the senescence associated genes *SAG12* (*At5g45890*), *WRKY6* (*At1g62300*), and *BFN1* (*At1g11190*) were analyzed. The transcript levels of *SAG12*, *WRKY6,* and *BFN1* showed a reduction of 72%, 69%, and 32% in the *ZmOSCA2.4* transgenic *Arabidopsis*, respectively, based on the delayed senescence observed in L1-L4 lines (Figure 9B).

### 2.12. Drought Stress and Rewatering Transcriptome and Small RNA Sequencing Predicted the Regulation of ZmOSCA2.4 Gene by miR5054

**Figure 10:**
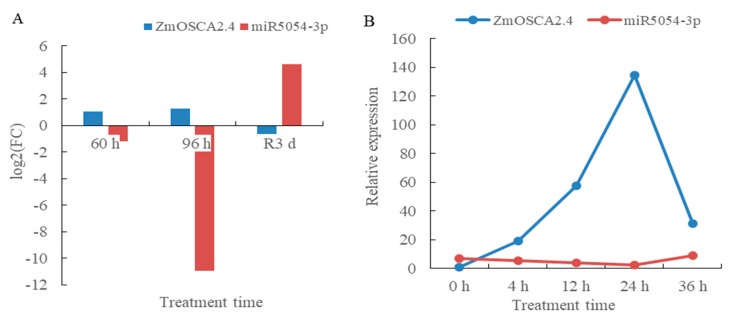
*ZmOSCA2.4* was regulated by miR5054 under drought stress and rewatering. (**A**) *ZmOSCA2.4* and miR5072 expression patterns in transcriptome and small RNA sequencing. The leaves of the three-leaf stage were stressed for 60 h and 96 h by PEG, and rewatering for 3 days denoted as T60, T96, and TR3d, and the control groups were named CK60, CK96, and CK3d, respectively. Quantification of *ZmOSCA2.4* and miR5072-3p expression levels were estimated using FPKM and TPM values, respectively. The FC in log2FC.CK60 vs. T60 is a fold change, which is the ratio of the expression between the CK60 sample and T60. It is log2FC after taking the base 2 logarithm; the same as log2FC.CK96 vs. T96 and log2FC.CK3d vs. TR3d; (**B**) relative expression of *ZmOSCA2.4* and miR5072 under PEG treatment at 0 h, 4 h, 12 h, 24 h, and 36 h. Data represent at least three independent experiments using RNA extracted from leaves of PEG treatment at 0 h, 4 h, 12 h, 24 h, and 36 h.

Preliminary transcriptome and small RNA sequencing analysis showed that miR5054 regulated *ZmOSCA2.4* gene. Under drought stress, the expression of *ZmOSCA2.4* was up-regulated and down-regulated after rewatering, as opposed to miR5054 (Figure 10A). Quantitative real-time PCR analysis showed that there was a significant negative correlation between the expression levels of miR5054 and *ZmOSCA2.4* at different time points under drought stress. Under drought stress, *ZmOSCA2.4* was significantly up-regulated while miR5054 was down-regulated (Figure 10B). There was a negative correlation between the expression levels of *ZmOSCA2.4* and miR5054, suggesting that miR5054 may indeed regulate *ZmOSCA2.4* gene, which also showed that miR5054 may play an important role in drought stress. We speculate that during osmotic stress response, there may be a depression of miR5054 to promote *ZmOSCA2.4* expression, and thereby stress resistance.

## 3. Discussion

As an important food, feed, and industrial raw material, maize is often exposed to abiotic stresses such as drought, high temperature, and high salinity results in reduced or even no yield. However, plants respond to adversity by regulating gene transcription and activating multiple mechanisms to reduce water loss and osmotic pressure damage, thereby sensing, responding to, and adapting to adversity. Previous studies of *Arabidopsis* and rice have shown that OSCA family members contain DUF221 domain and have been identified to contain 15 and 11 members, respectively, with multiple genes associated with osmotic stress [13,26]. Each OSCA protein in the *Arabidopsis* and rice genomes contained 11 transmembrane domains [27,28]. In contrast, nine ZmOSCAs contained 9–11 transmembrane domains, and ZmOSCA1.1a and ZmOSCA3.1 proteins contained only four and two transmembrane domains, respectively (Table 1), which indicates that ZmOSCAs had experienced greater genetic variation during evolution. Phylogenetic tree (Figure 2) analysis revealed that OSCAs can be divided into four classes, which was consistent with evolutionary analysis of *Arabidopsis*, rice, poplar, and grapes [13]. Each class included *OSCA* members from the monocotyledons sorghum, rice, and maize, and the dicotyledon *Arabidopsis*, which indicated that the *OSCA* gene family originated and diversified prior to the divergence of monocotyledons and dicotyledons. The classes III and IV of OSCA family contained few members, but have been preserved during species evolution, indicating they may perform an important role in a biological process.

On the basis of OSCA family phylogenetic relationships (Figure 2), protein structural domain (Figure 3A), and conservative motif analyses (Figure 3B), the maize OSCA family was highly conserved. The conserved motifs of member proteins in classes I and II were highly conserved, and composition patterns of conserved motifs in these two classes were highly similar in maize. In *Arabidopsis* [29] and animal cells [30], most of OSCA genes responded to ABA and salt stress, respectively, which was consistent with results of this study. However, we also found ZmOSCA3.1 contained fewer domains and conserved motifs than the other ZmOSCAs, and it showed little or no difference in expression level under PEG-, NaCl-, and ABA-induced osmotic stresses, suggesting that *ZmOSCA3.1* may not have a direct function in osmotic stress response.

Analysis of promoter components of the 12 *ZmOSCA* genes revealed that the other genes contained ABRE core components except *ZmOSCA4.1* (Figure S1). Classes I and II of the *OSCA* family also contained variable numbers of core components associated with drought, salt, cold (DRE core and MBS), and oxidation resistance (ARE), whereas classes III and IV contained only components that were resistant to oxidation and low temperature (LTR), respectively. The ABA response element (ABRE) is involved in ABA-dependent gene expression. Similarly, dehydration response elements (DRE) play a crucial role in ABA-independent gene expression in response to osmotic stress [7]. The ABA synthesized in response to water deficiency effectively inhibits opening of stomatal pores and promotes closure of stomatal pores to prevent water loss. In addition, ABA-activated gene expression is associated with plant adaptation to drought, such as the genes RD22, KIN1, and KIN2 [8]. The promoters of *ZmOSCA* members classified in the same group also contained different numbers and types of functional response elements, thus different genes classified in the same group may exhibit functional diversity. Taken together, these results suggested that genes in the same class may have different mechanisms of action and that synergistic effects may exist between genes in different classes [31,32].

Although the relationship between *OSCA* genes and stress response has been reported previously [17], the dynamic expression pattern of *ZmOSCA* and the relationship with osmoregulation remain unclear. Analysis of the relationship between *ZmOSCAs* expression patterns and osmotic adjustment will aid in understanding their function in osmotic stress response and provide a sound basis for future functional studies. As members of the OSCA hyperosmotic calcium channels proteins family, the majority of *ZmOSCA* genes responded to drought, salt, and ABA stress, consistent with the *OSCA* family members in rice [26]. The present results showed that *ZmOSCA3.1* was significantly down-regulated at 12 h under PEG stress, the expression level was unchanged under ABA treatment, and was significantly up-regulated at 36 h under NaCl stress, whereas the other 11 *ZmOSCA* genes were significantly up-regulated under the three stresses (Figure 5), suggesting that the 11 *ZmOSCAs* may be crucial mediators of drought stress response. Under NaCl stress, up-regulation of the other *ZmOSCA* genes (except *ZmOSCA2.5*) ranged from 10 to 100-fold, which indicated that these genes responded positively to salt stress. *ZmOSCA1.2*, *-1.3*, *-2.3,* and *-2.4* responded strongly to NaCl, PEG, and ABA stress, and showed peak expression levels 40 to 200-fold that of the control (0 h). Thus, *ZmOSCA1.2*, *-1.3*, *-2.3,* and *-2.4* were expressed simultaneously in response to ABA, PEG, and NaCl stress-response pathways. The results showed that there may be interaction and commonality in the pathways responsive to the three stresses. Regardless, it was evident that these genes played an important role in drought resistance and high-salinity tolerance and may have a conserved function.

Salt and drought initially cause osmotic stress to the plant [25]. Plants can reduce the osmotic potential by increasing solute concentrations, preventing massive loss of water, and maintaining cell turgor pressure, thereby maintaining normal physiological processes such as metabolism, stomatal opening, and photosynthesis [33]. As an important osmotic adjustment substance, proline can promote the transfer of Na^+^ into the vacuole. Under high salt stress, salt tolerance is enhanced by proline accumulation [34]. No previous studies have reported a certain relationship between *ZmOSCA* genes and osmotic adjustment substances. In the present study, we analyzed the correlation between the expression levels of 12 *ZmOSCA* genes and leaf proline content under ABA-, NaCl-, and PEG-induced osmotic stress. *ZmOSCA2.3* and *ZmOSCA2.4* were significantly related to proline content, with correlation coefficients higher than 0.9 (Figure 6). Under the three stresses, the peak expression level of these two genes was about 200-fold that of the control, which indicated that *ZmOSCA2.3* and *ZmOSCA2.4* may play important roles in the regulation of plant osmotic potential.

Protein interaction networks are composed of individual proteins that interact with each other to participate in all aspects of life processes, such as biosignal delivery, gene expression regulation, energy and material metabolism, and cell cycle regulation. String predicts the interaction between proteins using experimental data, information from other databases, and bioinformatic methods [35]. Protein-interaction assays showed that ZmOSCA2.3 and ZmOSCA2.4 interact with ZmEREB198, but not with EREB134 (Figure 7). These results differed somewhat from the STRING predictions, indicating that while STRING predicts potential interactions between proteins, the interaction requires experimental verification. On the basis of analyses of protein domains (Figure 3), expression patterns under the three osmotic stresses (Figure 5) and correlation between proline content (Figure 6), we speculated that *ZmOSCA2.3* and *ZmOSCA2.4* may have identical functions and mechanisms of action. ZmEREB198 is a member of the ERF transcription factor family and contains the AP2/ERF domain. Extensive analyses have elucidated the essential involvement of *AP2/ERF* genes in plant growth, development, and stress responses [36,37,38,39]. The present results suggested that ZmOSCA2.3 and ZmOSCA2.4 interact with ZmEREB198 to alleviate cellular damage in plants subjected to osmotic stress. However, no previous studies of ZmEREB198 in maize have been reported, thus exploration of molecular mechanism of ZmEREB198 interaction with ZmOSCA2.3 and ZmOSCA2.4 is worthy of investigation.

Previous studies have shown that *MYB44* (*At5g67300*) was associated with stomatal closure and tolerance to abiotic stress [40]. *DREB2A* (dehydration response element binding protein 2, *At5g05410*) can improve drought resistance [41]. *NCED3* (*At3g14440*) encodes 9-cis-epoxy carotenoid dioxygenase, an essential enzyme in ABA biosynthesis, which was regulated in response to drought in *Arabidopsis* [40]. *SAG12* (*At5g45890*) codes for a cysteine protease, and *SAG12*-specific mRNA levels increased with age [42]. *WRKY6* (*At1g62300*) codes for a transcription factor, and its expression markedly increased with senescence [43]. During senescence, the gene expression of *BFN1* (a bifunctional nucleases, *At1g11190*) was increased [44]. We analyzed drought tolerance performance in the different *ZmOSCA2.4* transgenic *Arabidopsis* (L1-L4) lines and found that drought tolerance was increased in the *ZmOSCA2.4* transgenic lines (Figure 8D). Compared with the wild type (Col-0), we also found the transcript levels of SAG12, WRKY6, and BFN1 decreased in the *ZmOSCA2.4* transgenic *Arabidopsis*, which explained exactly the reason why the chlorophyll content in transgenic *Arabidopsis* lines were higher than that of WT (Figure 8E). Combined with the increased expression levels for *MYB44*, *DREB2A* and *NCED3* (Figure 9A) in *ZmOSCA2.4* transgenic lines may explain why *ZmOSCA2.4* transgenic lines exhibit distinct drought-tolerant phenotype.

Small RNA sequencing revealed that miR5054 and *ZmOSCA2.4* had binding sites. In combination with high-throughput sequencing, a negative correlation was found between the two. Subsequently, we found that at different time points of drought stress, there was a negative correlation between the two expression levels. We initially speculated that *ZmOSCA2.4* was a target gene of miR5054. The function of miR5054 has not been reported. Combining *ZmOSCA2.4* can enhance the drought resistance of *Arabidopsis*. We speculate that miR5054 may also play an important role in plant drought tolerance and is worthy of further research.

## 4. Materials and Methods 

### 4.1. Plant Materials and Stress Treatment

Maize inbred line Yu 882 was selected as the material. The seeds were grown in Hoagland’s nutrient solution, which was refreshed every 2 days under long-day conditions (14-h day/10-h night with 60% relative humidity) in greenhouse. The leaves of three-leaf stage were treated with 20% PEG 6000 for 60 h and 96 h, and rewatering for 3 d, denoted as T60, T96, and TR3d, and the control classes (Hoagland’s nutrient solution) were named CK60, CK96, and CK3d, respectively. The above samples were sequenced by transcriptome and Small RNA sequencing. We also transferred separately the leaves of three-leaf stage to nutrient solution containing 20% PEG 6000, NaCl (200 mmol L^−1^), and ABA (5 μmol L ^− 1^). The leaves were collected at 0 h, 4 h, 12 h, 24 h, and 36 h and stored at −80 °C.

The *ZmOSCA2.4* was cloned into pFGC5941 vector using specific primers (Table S7). The constructs were then transferred into *Arabidopsis* by floral dip method. The presence of *ZmOSCA2.4* was verified by PCR using specific primers (Table S7). Four independent homozygous lines, L1, L2, L3, and L4, were selected. Seeds of wild-type and independent lines of *ZmOSCA2.4* transgenic *Arabidopsis* (L1-L4) were germinated on MS medium. Seedlings were then transferred to MS medium containing 200 Mm mannitol concentration in the vertical position. A soil mix consisting of sand, compost, and perlite (3:3:1, respectively), was weighed to standardize the quantity in each pot. After wild-type and transgenic *Arabidopsis* were watered regularly for 20 days, the plants were subjected to water restriction for 6 to 8 days, then rewatered for 3 days. The leaves of wild-type and transgenic *Arabidopsis* were collected at 6 DOD, 8 DOD, and R3D, then stored at −80 °C. All the *Arabidopsis* were grown in a greenhouse under 60% relative humidity with a 16-h/8-h day/night cycle.

### 4.2. Isolation of RNA and Quantitative Real-Time PCR (qPCR) Analysis

We extracted RNA from maize leaves treated with 20% PEG 6000, NaCl (200mmol L^−1^), ABA (5 μmol L^−1^) for 0 h, 4 h, 12 h, 24 h, and 36 h and the *Arabidopsis* leaves with drought stress for 6 DOD, 8 DOD, and R3D. Using a Hifair^®^ II 1st Strand cDNA Synthesis SuperMix (YEASEN, Shanghai, China) synthesized the first-strand cDNA. According to corresponding sequence, we designed gene-specific primers using Primer6, as shown in Table S7. *ACTIN 18s* and *ACTIN2* as internal controls for qRT-PCR in maize and *Arabidopsis*, respectively. The qRT-PCR analyses were carried out using Hieff^®^ qPCR SYBR^®^ Green Master Mix (YEASEN, Shanghai, China) on a Light Cycler 480 instrument (Roche, Basel, Switzerland) based on the manufacturer’s instructions. Each gene analyzed three technical replicates. The relative expression level (2^−∆∆Ct^) in the control plants without treatment was normalized to 1. The expression of miR5054 was determined using the TAKARA reverse transcription kit (TaKaRa, Shiga, Japan), using the miR5054 sequence as forward primer and universal sequence as the reverse primer for qRT-PCR.

### 4.3. Transcriptome Co-Expression Network Module Construction and Association Analysis Module and Proline Content

The drought stress and rewatering transcriptome resulting reads were aligned to the *Z. mays* genome (B73V3 Version) that was retrieved from NCBI. The transcriptome data have been deposited to the sequence read archive (SRA) under the accession number PRJNA477643. On the basis of reference genome, the mapping reads were assembled using Cufflinks software [45]. Quantification of gene expression levels were estimated using FPKM values.

The step-by-step method in the “WGCNA” package of R v3.5.1 software was used for co-expression analysis of drought stress and normal water-treated samples. According to the FPKM values of all genes in the sample, the soft-threshold of the co-expression network clustering was selected with R2 > 0.9 as the standard [46]. All FPKM values were converted into topological overlap matrix (TOM), hierarchical cluster analysis was performed on each gene, and different genes were classified into different co-expression modules by dynamic tree cut method [47]. All parameters were set as defined except “soft power = 14, min_module_size = 30, ME_miss_thread = 0.2”. For the results of gene co-expression analysis under drought stress, combined with proline content, the correlation between different co-expression modules and proline content was calculated through correlation analysis, and the correlation between gene expression and proline content was calculated.

### 4.4. Identification of OSCA Protein-Coding Genes in the Maize Genome 

The conserved OSCA domain DUF221 (Pfam accession number: 02714) was identified from the Pfam database [48] for constructing a search based on Hidden Markov Model-based searches (http://hmmer.janelia.org/), scanning against the maize genome (genome assembly: AGPv3) and the sorghum genome (genome assembly: V3). All the retrieved sequences were determined by the NCBI Conserved Domain Database (www.ncbi.nlm.nih.gov/ Structure/cdd/wrpsb.cgi) [49]. To determine whether the proteins contained DUF221 domain or not. The ProtParam (http://web.expasy.org/protparam/) was used to predict molecular weight (MW) and isoelectric point (PI).The transmembrane region (TMs) of ZmOSCA proteins were predicted by TMHMM Server V2.

### 4.5. Gene Structure, Additional Conserved Motifs, and Cis-Elements in the Promoter Regions of Abiotic Stress-Responsive Analysis of ZmOSCA Genes

We used phytozome v10.0 to download protein sequences of the identified maize and conserved OSCA domain Calcium-dependent channel (PF02714), Cytoplasmic domain (PF14703), and Late exocytosis (PF13967).The motifs of maize OSCA proteins were queried using online software Multiple Expectation maximization for Motif Elicitation (MEME) program (Available online: http://meme-suite.org/tools/meme) (Table S5).

To predict cis-acting regulatory DNA elements (cis-elements) in promoter regions of *ZmOSCA* genes, the PLACE website (Available online: http://www.dna.affrc.go.jp/PLACE/signalscan.html) was adopted to identify putative cis-elements in the 2000 bp genomic DNA sequences upstream of the Start codon (ATG) (Table S5).

### 4.6. Interspecies Microsynteny Analysis

In order to detect homogenous region between maize, sorghum, rice, and *Arabidopsis*, multiple sequence alignment was used to detect protein sequences of maize, sorghum, rice, and *Arabidopsis*, with the similarity of more than 70% (Table S6). Subsequently, the collinear block was then detected by MCScanX and the related downstream tools using the default parameters. Finally, the relationships of OSCAs orthologous genes among the four species were plotted using Circos software (Available online: http://circos.ca/).

### 4.7. Determination of Chlorophyll and Proline Content 

In brief, chlorophylls in leaves were extracted with 80% acetone, and the extract was incubated at 4 °C for 2 h in darkness. Chlorophyll content was assayed by measuring absorbance at 645, 652, and 663 nm with a spectrophotometer. The free proline (Pro) content in fresh leaf samples (0.5 g) was measured by the ninhydrin method. We Extracted the reaction mixture with 5 mL toluene, cooled it to room temperature, and read the absorbance at 520 nm [50].

### 4.8. Predicting the Interaction of ZmOSCA Proteins

The protein sequences of 12 ZmOSCAs were predicted by the STRING website (https://string-db.org/). The interaction coefficient between the proteins was greater than 0.5 (*p* > 0.5) (Table S4), and then poured into the Cytoscape software.

### 4.9. Validation of Interactions between Proteins using Yeast Two-Hybrid and BIFC 

The coding sequences of *ZmOSCA2.3* and *ZmOSCA2.4* genes were cloned into the yeast two-hybrid vectors pGBKT7 (bait vector), and *ZmEREB134* and *ZmEREB198* genes were cloned into pGADT7 (prey vector), respectively. Subsequently, the pGBKT7-ZmOSCA2.3 + pGADT7-ZmEREB198 were introduced into yeast strain Y2H using the high-efficiency lithium acetate transformation procedure [51]. PGBKT7-ZmOSCA2.4 + pGADT7-ZmEREB198, pGBKT7-ZmOSCA2.3 + pGADT7-ZmEREB134, pGBKT7-ZmOSCA2.4 + pGADT7-EREB134, which were also transformed into yeast H2Y by the same method, respectively. Primer sequences for pGADT7-T + pGBKT7-Lam (negative control), pGADT7-T + pGBKT7-53 (positive control), pGBKT7-ZmOSCA2.3, pGADT7-ZmEREB198, pGBKT7-ZmOSCA2.4, and pGADT7-EREB134 constructs are listed in Table S7.

The pSAT4-nEYFP-ZmOSCA2.3, pSAT4-cEYFP-ZmEREB198, pSAT4-nEYFP-ZmOSCA2.4, and PSAT4-cEYFP-ZmEREB134 vectors were constructed using gene-specific primers, respectively (Table S7). The constructs were then introduced into Arabidopsis protoplasts by PEG3350 penetration. Fluorescence microscopy was performed on a SP5 Meta confocal laser microscope (Leica, Germany). A yellow fluorescent signal indicates an interaction between the two proteins.

## 5. Conclusions

In this study, two co-expression modules related to proline content were obtained using WGCNA, including three *ZmOSCA* members. The phylogenetic relationships and domain structure revealed that 12 members of ZmOSCA family were placed in four classes (I, II, III, IV), all of which contained DUF221 domain. The promoter region of *ZmOSCA* genes contains multiple core elements responsive to abiotic stresses and hormones. A collinear analysis revealed that the *ZmOSCA* genes had diversified prior to divergence of maize. The majority of *ZmOSCA* genes were up-regulated in response to ABA, PEG, and NaCl treatment. In particular, the expression levels of *ZmOSCA2.3* and *ZmOSCA2.4* increased by more than 200-fold under the three stresses and showed significant positive correlations with proline content. Yeast two-hybrid and BIFC showed that ZmOSCA2.3 and ZmOSCA2.4 proteins interacted with ZmEREB198. Over-expression of *ZmOSCA2.4* in *Arabidopsis* remarkably improved drought resistance. Moreover, over-expression of *ZmOSCA2.4* enhanced the expression of drought tolerance-associated genes and reduced the expression of senescence-associated genes, which may explain why *ZmOSCA2.4* transgenic lines exhibit distinct drought-tolerant phenotype. We also found that perhaps *ZmOSCA2.4* was regulated by miR5054. These results provide novel molecular resources for breeding new drought-tolerant cultivars, and provide a reference for future analysis regulatory mechanism of *ZmOSCA* under abiotic stresses. 

## Figures and Tables

**Table 1:** Detailed information for twelve *ZmOSCA* genes in the *Zea mays* L. genome.

Gene Name	Gene Identifier	Chromosome	Protein Length (aa)	ORF (bp)	Number of Exons	Transmembrane Domain	Isoelectric Point	Molecular Weight (KDa)	Class
*ZmOSCA1.1a*	*GRMZM2G064189*	3	327	984	4	4	8.54	36.95	I
*ZmOSCA1.1b*	*GRMZM2G021194*	3	768	2307	11	9	9.05	87.51	I
*ZmOSCA1.2*	*GRMZM2G456000*	8	768	2307	11	9	9.15	87.93	I
*ZmOSCA1.3*	*GRMZM2G181206*	6	748	2247	11	10	9.15	85.65	I
*ZmOSCA1.4*	*GRMZM2G128641*	1	810	2433	11	9	8.66	93.88	I
*ZmOSCA2.1*	*GRMZM2G163059*	3	586	1761	8	7	9.35	66.76	II
*ZmOSCA2.2*	*GRMZM2G409093*	1	765	2298	10	9	9.3	87.38	II
*ZmOSCA2.3*	*GRMZM2G164470*	5	749	2250	10	10	8.8	86.15	II
*ZmOSCA2.4*	*GRMZM2G039186*	1	699	2100	10	9	8.74	79.04	II
*ZmOSCA2.5*	*GRMZM2G402708*	8	706	2121	10	11	9.05	79.64	II
*ZmOSCA3.1*	*GRMZM2G162253*	5	249	750	2	2	8.88	28.68	III
*ZmOSCA4.1*	*GRMZM2G059891*	9	796	2391	1	9	7.67	89.4	IV

## Supplementary Materials

Supplementary materials can be found at https://www.mdpi.com/1422-0067/21/1/351/s1.
