# Supplementary material for: Systematic Analysis of the Maize OSCA Genes Revealing ZmOSCA Family Members Involved in Osmotic Stress and ZmOSCA2.4 Confers Enhanced Drought Tolerance in Transgenic Arabidopsis
Source: Int J Mol Sci. 2020 Jan 5;21(1):351. doi: 10.3390/ijms21010351 (PMC6982122; doi:10.3390/ijms21010351)
Supplement: Supplementary file 1 [file ijms-21-00351-s001.zip › ijms-680479/Figures S1 and S2.docx]

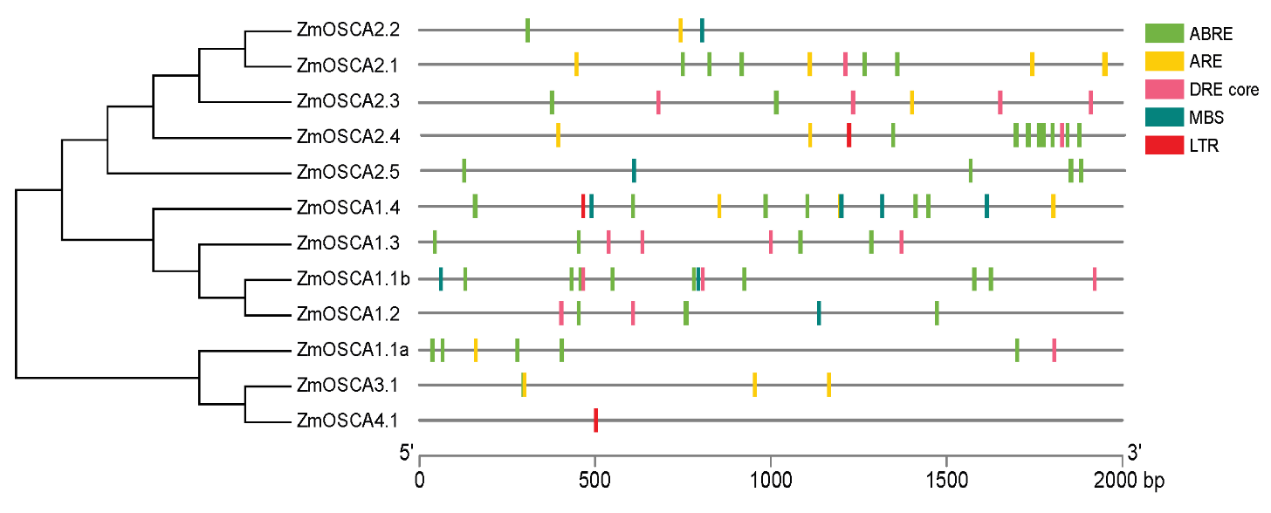


Figure S1.Cis-elements in the promoter regions of maize ZmOSCA genes.The stress-responsive cis-elements are distributed on the grey line. The ABRE, ARE, DRE core, MBS and LTR sequences are respectively represented by rectangles of different colors. ABRE: ABA and drought responsive element; ARE: antioxidant responsive element; DRE core: Drought, salt and cold responsive element; MBS: dehydration-responsive element; LTR: low temperature responsive element.


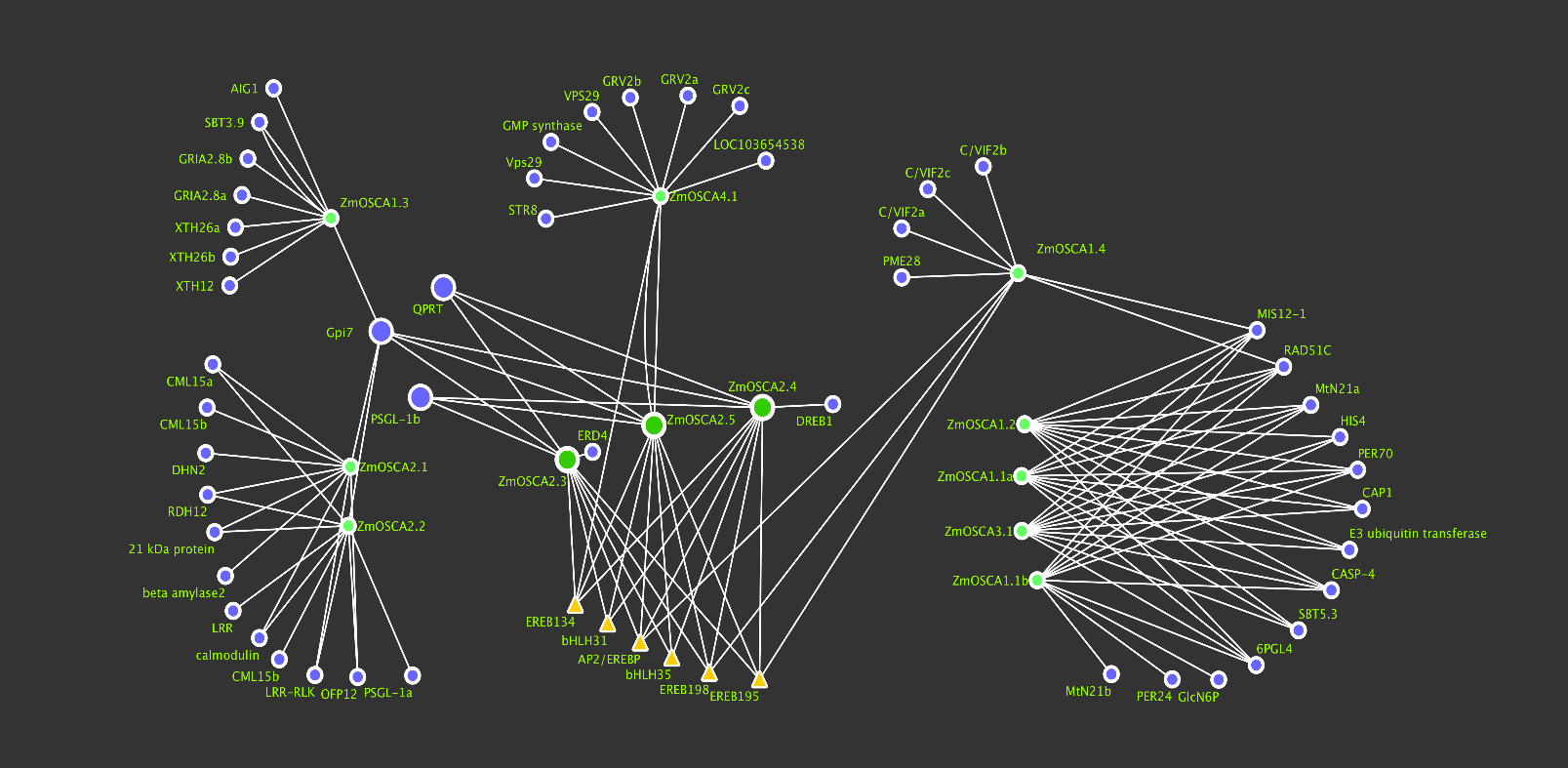


Figure S2. Interaction network analysis of ZmOSCA proteins. The Cytoscape software was used to map the interaction network between12 ZmOSCA proteins and potential interaction protein (p > 0.5). The white line indicates an interaction between proteins. The green circles represent the ZmOSCA members and the blue circles represent the proteins interacting with the ZmOSCA proteins, wherein the interaction transcription factors are represented by orange triangles. ZmOSCA with the same interaction transcription factor are shown in large green circles.


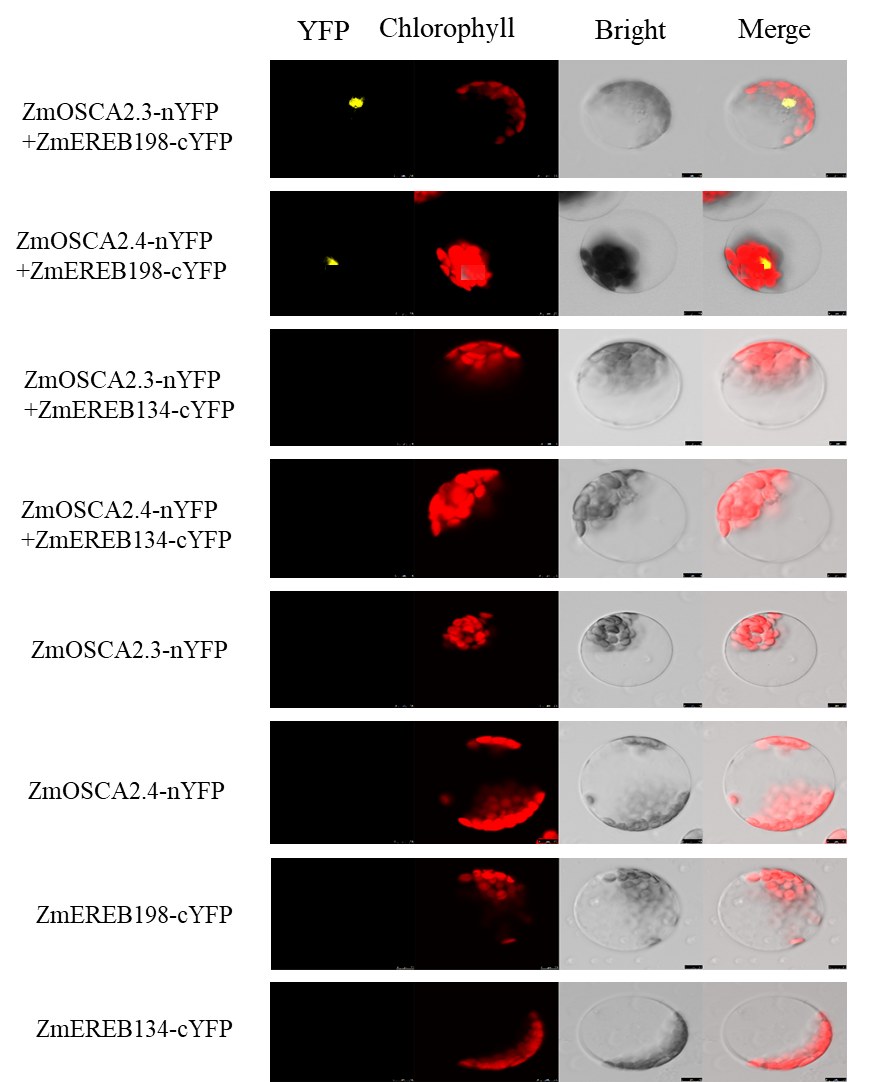


Figure S3. The bimolecular fluorescence complementation analysis for interactions between ZmOSCA2.3, ZmOSCA2.4 and ZmEREB198, EREB134 proteins. The nYFP and cYFP represent the pSAT4-nEYFP and pSAT4-cEYFP vectors, respectively. The stronger the interaction is, the stronger the signal will be. Scale Bars = 8 µm.
