# Supplementary material for: Systematic Analysis of the Maize OSCA Genes Revealing ZmOSCA Family Members Involved in Osmotic Stress and ZmOSCA2.4 Confers Enhanced Drought Tolerance in Transgenic Arabidopsis
Source: Int J Mol Sci. 2020 Jan 5;21(1):351. doi: 10.3390/ijms21010351 (PMC6982122; doi:10.3390/ijms21010351)
Supplement: Supplementary file 1 [file ijms-21-00351-s001.zip › ijms-680479/Table S5.doc]

>ZmOSCA1.1a

MFMSKVEGLTSISSLERRSASKYYIFIFFNVFLASIIAGSALEQLKSYIHQSANEIPRTIGEAIPMKATFFITYTMVDGWAGVAGEILRLKPLVIFHLKNFFLVKTEKDREEAMDPGSIGFDSSEPQIQLYFLLGLVYAAVTPFLLPFVLIFFGFAYVVYRHQIINVYNQEYESAAAFWPSVHGRIITALIVSQLLLLGLLSTKGAGQSTPVLLVLPVVTFYFHKYCNNRYKPTFVKCPLQEAMKKDTLERAREPGFDLKGYLMNAYIHPVFKGDGDDDKFSVVDQPEAEQVLVATKRQSRRNTPVPSKYNGSESPSLPEIVNDQRL*---------------------------------------------------------------------------------------------------------------------------------------------------------------------------------------------------------------------------------------------------------------------------------------------------------------------------------------------------------------------------------------------------------------------------------------------------------------------------------------------------

>ZmOSCA1.1b

MATIQDIGLSAAINIMGAVLFLVAFAFLRLQPINDRVYFPKWYLKGARESPSHGGTFVRKFVNLDMRSYLKFLSWVPAALKMPEDELISHAGLDSAVYLRIYLVGLKIFAPISVLAFIVLVPVNWTNDTLQFSKLEHSNVDKLSISNIPVGSKRFIAHLAMAYVFTFWTCYVLLREYEVVAKMRLRFLSSEKRRPDQFTVLVRNIPPDPDESISELVEHFFLVNHPGHYLTHQVVYNANKLAKLVKEKAKMHNWLDYYQLRFERNASKRPTTKTGFLGCFGTKVDAIEYYTSEIERIENEEAEEREKIVKDPKSIVPAAFVSFRSRWGAAVCAQTQQTSNPTVWLTEWAPEPRDVYWDNLSIPFVSLTVRRLIIAVAFFFLNFFYVFPIAFVQSLANLEGIEKAAPFLKPLIEEHTIKSFIQGFLPGIALKIFLILLPSILMFMSKVEGLTSISSLERRSASKYYIFIFFNVFLASIIAGSALEQLKSYIHQSANEIPRTIGEAIPMKATFFITYTMVDGWAGVAGEILRLKPLVIFHLKNFFLVKTEKDREEAMDPGSIGFDSSEPQIQLYFLLGLVYAAVTPFLLPFVLIFFGFAYVVYRHQIINVYNQEYESAAAFWPSVHGRIITALIVSQLLLLGLLSTKGAGQSTPVLLVLPVVTFYFHKYCNNRYKPTFVKCPLQEAMKKDTLERAREPGFDLKGYLMNAYIHPVFKGDGDDDKFSVADEPEAEQVLVATKRQSRRNTPVPSKYNGSESPSLPEIVNDQRL*------------------------------------------

>ZmOSCA1.2

MASLGDIGLAAGLNVLTAVAFLLAFAFLRLQPINDRVYFPKWYLRGMRDNPIVSGAAVQKFVNLDARSYLKFLNWMPAALKMPQDELINHAGLDSVVYLRIYVTGLKIFVPITVLAFLVLVPVNWTNDTLGRINVVYSPIDKLSISNVPNGSKRFIAHLGMAYAITFWTCYVLLKEYEIISNMRLRFLASEKRRPDQFTVLVRNIPPDPDESISELVEHFFLVNHPDHYLQHQVVYNANKLADLVEKKKKMRNWLDYYLLKYERNSSLRPTTKTGFLGCFGSKVDAIDYYKSEIEKIGKQEAEERKNVMKDPKSVVPAAFVSFRSRWGAAVCAQTQQTSNPTLWLTEWAPEPRDVYWNNLSIPFVSLTIRRLIVAVAFFFLNFFYVIPITFVQSLANLEGIEKALPFLKPLIDLPFIKSFIQGFLPGIALKIFLIVLPTILMFMSKFEGLISQSSLERRSASKYYIFLFFNVFLGSVIAGSALEQLKTYLHTSANDIPRIIGDSIPMKATFFITYVMVDGWTGIAGEILRLKPLIFFHLKNFFLVKTEKDREEAMDPGSICFDSTEPRIQLYFLLGLVYAAVTPFLLPFILVFFGFAYVVFRHQIINVYNQQYESAAQFWPSVHGRIITALIVSQLLLLGLMSTNDFEQTTPVLLVLPVLTFGFYKYCKNRFEPAFVRNPLQEAMKKDTLERAREPNLDLKAYLANSYLHPVFKGDEGDDRYSVMDDDGWMEEEVIVPTKRHSRRTTPAQSKHEGSDSLSVLESSLQR*------------------------------------------

>ZmOSCA2.5

MDAEGLLASAAINLGLALVALSLFSLLKKQPGNAPVYRPRRMATGDLRADGGFLPLGHGRLTPSFRWIGAAFRLSEDDVLRRHGLDALVIVRLFKFGINCFSVCSIVGLLVLAPTNYTSEGRAEIRRSNSMELFTVTNVTRGSNRLWVHYSCLCFISLYVVYLLHKEYREITMRRIEHLKHHYKRYDQYTVLVRGIPTCPDHGAYGCYVDHFFSKHYRTYRSYQIVHDIGNIEALQKLASSIEKRIQRKRETSKCSLLGRIWSKFTSDATGIHNHEEKLKNVQDTIRLLQCENMLKQKEVPVAFVSFKSRLEAAQAAETQQLANPLSLVTTYAPEPTDTIWKNLSIPFWRMATYRLGVFAAAVLLTVFFTIPVTAVQGIAQFEKMKRWFPPARAVQLIPGLNSVVTGYLPGMILNGFIYLVPSAMLGMASFEGCVASSQREIRACDMVFYFLLGNVFFLSVLSGSLLDQIGESFTHPKNIPNRLASAVSAQSDFFITYILTNGMSGFSLEVLQFGLLIWQFLKARSIGHSEEPYLYGFPYFRVVPVVSIAILIGVVYAVVAPLLLPILLVYFLLGYAVYINQMEDVYEITYDTCGQYWPKIHHYIFLSIALMQVTMIGLFGLKSKPGASLATVPLLVLNILFNEYCKARFLPTFRHRPVQVRRAANELDEAEGTTEGDVDDAICAYRPPWMRPTNLESSSVEPLNV*--------------------------------------------------------------------------------------------------------

>ZmOSCA3.1

MVERSKSYRSLVRVCFTFLKTNECHFGYCPNTFYRSMVVTDHTKADKIYQEIEGHKQKIARAEVVYANSKTESNTEGTRPTHRTGFLGLIGTKVDTIEYCSEQIKELLPKLEAEQKTTLHEKQQRAAIVIFNSRSAVAFASQTLHAQVYDKWTVMEAPEPRQIIWSNLPMKLYDRQIRQSVVYGIVFLIVVFYMVSLTAIFAVTTLENLEKLPFLKPVVEQPAIKTVLEAYLPQIAPIICHMILLLFLF*---------------------------------------------------------------------------------------------------------------------------------------------------------------------------------------------------------------------------------------------------------------------------------------------------------------------------------------------------------------------------------------------------------------------------------------------------------------------------------------------------------------------------------------------------------------------------------

>ZmOSCA2.3

MEFSALLTSAGINIAVCVLFLSLYSILRKQPHNFSVYFGRRLAEERFQRQDDYFSFERLLPTAGWIVKAYWCTEEEIRQVAGLDSVVFLRLFIFSIRIFSITSLVCVFVVLPVNYHGKEMNHNHIPEESLNVFTIANIVEESRKLWVHCSALYVITISACILLFHEYKYISRKRLAHVTGYPPNPGLFTVLVRSIPRFDNELLDDTIRNFFVNYHGSSYLSHQMIFRKGHFQKFVDRAERAYRRFVRVRLSVSERNGRSSMSRCGVCGVRASSFQLYRNKFIEAKKSDLTNPEVVEAQKNCPGALVFFKTRYAAVVASRVFQSSNPMLWVTDLAPEPRDVYWSNLWIPYRQIWLRKLATLAASIVFMFVFIVPVAFVQSMMQLEQLKRMFPSLTGLLNKSFFARVITGYLPSVTLLLSLYTVPPLMMLFSSIEGSISRSGRKRSGCLKILFFNIWNVFFVNVLSGSVLNQLNVFSRPKDMPTMLAELVPKQATFFITYVLTSGWASLCSEILQVFNLVYNFFSRFVCCCHRQNKEYVYSFPYHTEVPKVLLFNLLGFTFSIMAPLILPFLLVYFCLGYLVYRNQILNVYYPKYEMGGKLWPVMHNAIVFSLVLTQVIALGVFTIKRSPVTAGFTILLLVGTVLFNEYCRHRFSRIFEAYSAQDVIELDRDDEQSGRMQEIHQHLLDAYSQTPPGEGSSRGGSQVPIELIMEDPAQEASESSQELCDTVQEVSGAIHDHDHVVEDKAHSV*-------------------------------------------------------------

>ZmOSCA1.4

MATLEDLGVSAFINILGAFVFLLLFAVLRIQPVNDRVYFPKLYLAHKRHQHDHAARSAFRRFVNLNLCTYVTFLSWVPGALRMSEPDLVAHAGLDSAVYLRIYTLGLKIFLPITTLALLVLIPVNVSGGTLLDLRKEVVFSDIDKLSISNVSPGSNRFFIHLLMAYVFTFWVCFMLYKEYSNVAFMRLHFLASQKRCADHFTVIVRNIPRVSSHSTSETVDEFFRRNHPDHYLGHQPVYNANRYAKLVKQKERLQNWLDYYELKFERHPERRPTRRTGCLGFCGREVDQIDYYRARISELERRMTSERQKILNDPKAVMPVSFVTFDSRWGAAVCAQTQQSKNPTQWLTDWAPEPRDVYWQNLAIPFFSLSIRRFLMSVAVFALVFFYMIPIAFVQSLANLEGLEKVAPFLKPVIEAHVVKSFLQGFLPGLALKIFLYILPTVLLIMSKVEGYVSLSSLERRTASKYYYFMLVNVFLGSIIAGTAFEQLYSFLHQPPTQIPRTIGVAIPMKATFFMTYVMVDGWAGIANEILRVKALVIYHLKNMFIVKTERDRDRAMDPGSIGLGENLPSLQLYFLLGLVYAVVTPLLLPFIIVFFAFAFLVYRHQIINVYNQEYESAAAFWPQVHSRIIASLLISHVTLFGLMSTKEAAYSTPLLIFLPLLTIWFHKYCKSRFEPAFRKYPLEEAMAKDTMEHASEPNLNLESFLANAYLHPIFRLFEEAGKEEETVEVRIDKAEKQQHRHEEDEAHARSSSQYREEETHFRSTHETHYHHEESHVSRSTQYHEGIHVRSDTDSPSPPHFVYHYDIQQ*

>ZmOSCA2.2

MKVGGLLTSAGINIGLCVLFLSLYSILRKQPQNVKVYFGRRIAEEHNRLRDAFILERFVPSPSWIVKSLRCTEEEILATAGLDAVVFNRILVFSIRIFSLAAILCVFGVLPLNYFGQDMHHVRIPSASLETFTIGNVEERSRWLWVHCVVLYIISAVACILLYLEYKHIARLRLYHISRATSNPSHFTILVRGIPKSSTESFSRTVESFFTKYHASSYLSHQVVYKVGKVQKIVSGAKKVYRKFRHFKGATVDRRCRPIRFQCCFCGASSNSFQLLPSDYEQESEKSDVNESSSSLPDEECGAAFVFFKTRYAALVVAKILQTSNPMRWVTTLAPEPDDIYWSNLWLPYKQLWIRHIVTLMCSIVFMVVFLIPVTFIQGLTQLEQLQQRLPFLRGILKKKYMTQLITGYLPSVILQIFLYTVPPTMMFFSTLEGPISHSERKKSACCKVLYFTIWNVFFVNVLSGSAISQLNALSSPKDIPMQLAKAVPVQATFFTTYVLTSGWASLSSELMQLFGLTWNFLRRYLLRIKEDSDFLYSFPYHTEMPKVLLFGLLGFTCSVLAPLILPFLLLYFFLGYVVYRNQFLNVYCTKYDTGGQYWPIAHNTTIFSLILTQIICLGVFGLKESPVAAGFTVPLIIFTLLFNQYCRKRHLPLFKTFPAQNLIDMDKEDQQSDTTEDLHERLHSAYFQFPDTDEVPLERVRVSVGRDEDGSGSSGESSRKESAEDEHKSGLSHPTLDGLPVSRLRNAVRSLGSVLRPQRRQLPV*---------------------------------------------

>ZmOSCA2.4

MILSALTTSVGINLALTVLLAAAYSLLRRRPPYVEVYSPRRPYAPLEPWLAAAWRRAEEDIHAAAGLDGVVFIRIFVFSIRVFAAAAVLGVGVLLPVNFLGDQLREIDFTDLPNKSIDLFSISNVQDGSSKLWLHFSAVYIITGITCYLLYHEYKYISGKRLEYFMISKPLPQHFTVLVRAIPVSDGVSVGDAVDKFFKEYHASTYLSHTIVRQTGKLRRLLNDAESICTKLTNLNHVRRSTGDPPRKLGLFSRNDLVGEYQKRLEDLEENVRMEQSDATRRQEIPAAFVSFRSRYSAANAVYIRQSDNPTEWQTEEAPDPHDVYWPFFSTSFMERWIAKFVVFVASVLLILVFLLVVAFVQGLTYLEQLEKWLPFLRNILEIAVVSQLVTGYLPSVILHVLSSCVPSIMKLFSTMQGFVSVSGIEQSACNKMLRFTIWTAFFANVLTGSALVQFEIFLNPKELPSKLAVLVPAQASFFIAYVVTSWTSITSELTQITALFCHLWGKCAKCCKRDYSKAPSMPYYSEIPRILLFGLIGLAYFIVAPLILPFVLVYFCLGYFIFRNQLFNVYAPKYDTGGKFWPVVHNTTIFSLVVLHIIAIGVFGIKKLPLASSLLLPLPPLTLLFNEFCRNRFLPIFEAYSTESLLKKDREEQSKPDMAEFFSNLVTAYCDPALKPFQRASNSDERTAPLLASLSSSR*---------------------------------------------------------------------------------------------------------------

>ZmOSCA1.3

MGSLTDIGVSAGLNILSAVGFLLAFAVLRIQPINDRVYFPKWYLKGTRSSPRQLGNVFLKFVNADFSTYIRFLNWMPAALKMPEPELIEHAGLDSAVYVRIYLLGLKIFVPIALLAFAVLVPVNWTSGTLENEKGLSYDQIDKLSISNLGKGSKRFWAHIAMSYVFTFWTFFVLYHEYKVVTTMRLRFLANQNRRPDQYTVLVRNVPPDPDESVSEHVEHFFAVNHRDHYLSHQIVYNANHLSGLVEKKKGLQNWLIYYENKHAKNPAKRPKIKTGLWGLWGQRVDAIEYYQKEIEDLCKQEDEERQKVVNDPNYIMPAAFVSFKTQWGAAVCAQTQQTSNPTVWLTEWAPEPRDVFWANLAIPFVELSVRRLIVAVAFFFLTFFFMIPIAIVQSLANLDDIVKVLPFLKPIIERNSLKSVIQGFLPGIILKIFLILLPTILMAMSKIEGHTSLSGLDRKTATKYYIFLFVNVFLGSVITGTAFQQLDNFIHQSANKIPEVVGESIPMKAAFFMTYIMVDGWSGIAAEVLRLKALVIFHIKNAFLVRTEHDREQAMDPGSLDFYNCEPRLQLYFLLGLVYAVVTPMLLPFIIVFFSLAYLVFRHQIINVYTQHYESGAQFWPDVHMRLIIALIVSQILLLGLLSTQEAEKSTVALLPLPVLSIWFHYVCKGRFEPAFVKFPLQDAMVKDTLERAHDPTLNLREYLKGAYVHPVFQKNDIYKVIAVDEEEKNPMVVTKRQSRMNAPAGS*--------------------------------------------------------------

>ZmOSCA2.1

MKISALLTSAGINIGLCVLFLSLYSVLRKQPANVRVYFGRRIAEEHDRLRGAFILERFVPSTGWIVKALQCTEEEILAAAGLDAVVFNRILVFSIRIFSLAAVLCVFGILPLNYFGQDIHHVRIPSESLDIFTIGNVEVKSRWLWVHCVTLYIISAVACILLYIEYKHIARLKLLHLTSATPKPNHFTVLVRGIPKADKESCSDVVDNFFTKYHSSSYLFHQVVYKVGKVQKIMTGAKKAYKKFKHFTDETVDQGCRTVTYRCCLCGASSNSFKLLNTECEQNKGKADNKSILDLDDEECTTAFVFFKTRYAALIASEILQTSNPMKWVANLAPEPEDVYWSNLWLPYKQLWARRIATLLGSIFFMFIFLIPVTFIQGLSQLEQLQQRLPFLRGILKKKYYMTQLVTGYLPSVILQIFLYIVAPIMMLFSTLEGPTSHSERKRSACCKVLYFTVWNIFFANVLSGTVISQLNVLSSPKDIPVQLAKAVPGQATFFITYVLTSGWASLSSEVMQLFGLIWNFIIKYVLRMREDTEFVPSFPYHTEVPKVLLFGLLGFTFSVLAPLILPFLLVYFCLGYVVYRNQVRS*--------------------------------------------------------------------------------------------------------------------------------------------------------------------------------------------------------------------------------

>ZmOSCA4.1

MGPAAQPPDAGGGGDPEAWYGSIQYLINISAVGAASCVLLFLLVKLRFDHRRIPGPSALAAKLLAVYHATAAQIALHCGADAAQFLLFERASFLILAAVAAAAVAAALPLNLLAGDAAVVDQFASTTISHIPKSSPLLWLHLLLTAAVVAIAHLGISRMEDALRITRFRDGNGNPSDPNSSSVAVFTIMIQGIPKTLAADKTPLKDYFDHKYPGKVYRVIVPFDLCTLEHLVEQLGKVRNKISWLEARLGARDLFDDIVHDNDDTVQSEEHWFVRRCRGLWAMAGERLGFTDEERLRKLQTKKLVLASRLSDYKEGCAPGAGVAFVVFKDVYTANKAVKDFKMERKKTPIGRFFPVMELQLERSRWKVERAPPASDIYWNHLGLSKISSRLRRIAVNTCLILMLLFFSSPLAIISGMQNAARIINVEAMDHAKSWLAWLEGSSWFWTIIFQFLPNVLIFVSMYIIIPSVLSYFSKFECHLTVSGEQRAALLKMVCFFLVNLILLRALVESSLESWILSMGRCYLDGPDCKQIERYLSPSFLSRSSLSSLAFLITCTFLGISFDLLAPIPWIKHIMKKFRKNDMVQLVPEENEDYLPMQNGEETNNLTAPLMPEREDGGLLDNIEGHDLSLYPLNRSFHMPKQKFDFAQYYAFDITIFALTLIYSLFAPLVVPVGATYFGYRYLVDKYNFLFVYRVRGFPAGNDGKLMDRVLCIMQFCVIFFLAAMLLFFAVQGDSMKLQAICTLGMLVFYKLLPSRSDRFQPSLLEGMQTVNSFVDGPTDYEVFSQPDLDWSLYQS*--------------

> ZmOSCA1.1a

CCACGGCGCGGCCTCGTTCAGCCCCAAGGCCCACGTGTGATCTGGCCCATCGTAACGGGCCCCGCGTGGCCGCCTCTGTATTACGGGCCTAACTTGTAA
AGGCATACTTGTAATTACAGCTTGTAACCCTGCTTTATGGGAATATTCCGGGGATAAACCAGGCGTCTGAGGGCACATGCGTCCTTAACACAAGACGCTG
GGCACTCAGATACCTATAAATACCCCCGCACAGTGCCCGTGAGAGGCTAGATTAACAGAGCTATTGCCCCCGTGCACGTAACCCTGTTGTCACCGCTGTT
CACCCTAGTTGGCCTACTTGCAGCAGAGAGCAAGTTCCAACACACTGTTTCCTGTAGAGGGGACTTTAGTGCTGACTGCTGCACCTAAAAAACTACCCTA
CACGTGCACTGTGCAAGGGGAGGGGGCAGCTCGCCACACGCGCTGCGTACAGACTGTAAATGACGGGTATAAAGGATGAATATAAAGGACGCTGCTGGAG
ATAGCCTAATTATGTCATTGCACAAATGTAATGATTTAAATCATTTGTTTTGATAGAGTTGGATCATAGCCAAACTTTTGACACAAGTACACATTATATT
TCAAATATACACACATTAAGAAAAGTAAATTAAGCTTTTTCTGAAGTGGATTTTCAGTTTTTTAGTGATATAAAATGTGGAGGCTCCCACATAAGGGAGG
CAGGCTGTTTTGATCTTGCGACCTGGTGAGTCCCAGTGGCGGAGGATGCACTAAAATTGAGGTATTGCTAATGTAACAACGAAAGAAAAATTCACAACAC
AATCTATAATATTTCATAAGTAATTTATAGCTATAAAATAAGCAATAAATGTGAAAAAAATTATTCAAGAGTCATTGTGTCGATAATCCATCTACAACCA
AATATTATAAAGCAAGTAAAATCTGAAAAAAAGAGAACAAGATAATGATGGTAAATTGAAGTAAAAACGATATAAGAATATACCTCTAGTTTAATCTAGG
TCTAGAAGACCTAGACAATTGCATTTATATATTTTTCTTACTTTAAGATGTCTTTTTAATTTTATGAAGATCAAGTCCTTTTAAGATTTCTCTTTTGATA
TAGCACATCATCAAATCATTTAACTAATCATTGGTCATCTTGTTGCGTGGAGCTAGGCGGAGGTTGTTGTGTTGATTTGCTTTGAGCTCCTCGACGTTCT
AAGAACAGGGACGAGTGAATGGCGATAGGGGCTGCAATACAATAGGAACTGAAGCTGGACGATAGGCGGAATAGGGGTCAGGGGTGAATCGCAGAGCTAG
GTCAGGTAGAATCGCGCCGGACAGGTGGAGCTAGGCGATGGGCGAAGCGAGGGCAAACGACGAAGTGCGAAGCTAAGCTTGGATGGAGACACGCGGAGCT
GGCTGGGCCGCTGAGGCACGCCACACGCGGAGCTGGCGATTGGGCAGACGATGAAGGCCCCGTTTGTTTGGGTTTTTTTAGCTTCTGACACCAAAAGCTG
TTGCGGACTGCCAAACGCCCCTAGCTTTTCAGCCCACCTGTATAAGAATCGTTTTGGTAAAAAACATCCAAATCAACATGAACACAAAATCGGTCGAGTC
GTCACGATAGTAGGAATCTATCGCTTTCTAGATCCTAAACCCTTTGGACCACTTCATCTTCCTCCGCATTAATCCTCACGATACTCAGATTCTTCCCACG
TCCAGAATCTCTCTACAGCCAGATTCTCAGAAAAGCTGGTCAGCAAAAAAATTGAACCAAACAGGCCTGAAGGCTCGTGGAGCTAAGCGAAGGCAGTCCT
AGTCGGCTAGGAAGAATAGAGCGTGGGATAGAGATTTATTTTACTATTGGTATTTTATTGGGTTGAATCAAGGTATTGCTTGAGCAAGGCCGAGCAATAT
TGGGTCCTCCGCCCATGCTGGTGACCTAGTGAGTAGTGAGACAATTCTCACTACTGCACCAGGCCTGCCCTTCTTCATAAAATATGTTACTGAAGGAGCC

> ZmOSCA1.1b

TAGTTCTTACAGTACTTGTGTTAGTGGTGTGAGCTGCATCTTTTTTTTCTTGGATCAGTTGGCGTGGTGGCGCTCGATGACCGATCGATGCACATGCACC
TAATTCTAATTCGCGTTATCTGACCACGTACGCTAGATGCTTTGATGCTTTTGTTTCTCGTGCCGAATTATTTAGATAAATTAATGCGATGAACCTTCTC
GTCGTAACTTATTGTCAAAATCGTCCCCTCTTGTCCGGTTCGCCGAAGCAAGCTTCCTAAGCACATATCTCCGATTAACAAAAAATATGCTTCAAACACT
CGAAAACGTTAACCTAGCTAAGAATCCTCGGCCAAGACTCGCACCAACGGAATCAAACCTAGAATGATATTTATAAATAAACACTAAGAAGTTCATTTAA
TATTTGTATTTAAATGGAATACAAATACACGTAGTATCTATATTTTGACCGTCGCCACGCGTCGGCGGAGAGGGAGCTGCCCGGGGCAGGGCAGAGCACC
GCGCGTCCCGGCCTCTCGGGCTGCCACACCCGCACGGGCCACACACGTGTTTCGATTCGGATCCTTTTTCTTCTTTTTTTTATCAGCGTACGTATTTGAC
CTTGGGATGGGAGGGGAAGCCAGGCCAGGCGCTCTGGAGTCTGGGCTCCGGCTACCGCTGTCTTGTGTGTGGTGCGGAGACCGGAGAGGATTTGGACTAG
GGAAGGAAGGGCAGCGGCTGGCGGGTTTGGTGAGTCGGATCGGATGAGCACGGGCCGTCCCGTCCGTTGGAGGTCCCGGGTTCCGTGACAGTTGGGCGTT
GCCGACAGGCCGTACGAGACAGAGGATGGCGACCGCGATTCCTCTCAAACCTCGCCACGCCACGCCACAACAAACACGCGCCGATTAAATATCATCACTA
GTCTAGTCTCCCGTGACTCACGTATGCCTGATTGACTTTTGCCACGAATGTCCGTGTTTGTCTCGTCTTCTTTACCTTACCCCTCGCTTACTAAATCTAC
GCTTCACATAAGCTCTGCAGCTACAATCTATAAATATAAAATACTATATAAATAATATAAAAGTGGTCGAAGCTTTGATCAGCTAAGTATAGAAGGGTAA
CCGTGTATTTTTTTTATTCTGGACTTAATAACAATCAATTTATTCTACAGTCGACAGACACTCATTACGGCGGCATCAAGACATACCATCAAGGCTGCTC
TCACAGCATTCCGCGCCGGTAATACAAATGAAATGGCCCATGCAAATAAATAGTACTGTAATAAAAAGGGGTGGCGAAAAAGGTTTAGGGCTAGTTTGGG
AACACTAATTTCCTATGGGATTTTCATTTTGAAATTAGTGTTCCCAAACTAGCCCTTAGTGTTGGCAATGCAATCCATTTCCCGTCCGGCTCCAGCATAT
CTGCTGCCCGGCTGGCGGCTGGCGAGAATCGTAGTGGCGACCAGCGAAGCATACATGACAGGACGAACCAGCAGCGTCAGCTCTCTTCCCTCCCCGTCCC
CGCTCTCCACGCCTGCTGCCTGGGTGGGTATTTATATATTAGAGTGGTTGGTTAGGATCACATGTGGGACCCCCACGTCTCCTCCTCCGGATGGACGGGA
CGGGACGGACGCTTCCGGTTCACGTCTGGCCATTCCACGCACGCTTTCCCCTCTCTCTGCTGGCTCGTGCTCCGCTCACTCGCTCGCCATAAACGCGCAA
CAGCTGCAGGCAGATCTGTCCGTTACCGCTGCTGCTACTTCCTTGCTTGTATTTTATATGTAGGTGAGGTAAGCGGCCACGGAGCAAAGGAGGAGTGAAG
TGGTAACTGCTGCATAGCATCGATCCCTCTCTCTCTCTCTTCTTCCTCCCGTTAAACTCTGATCGACTGTCCAGCAACCCAGCCGTAACGTCTCCTTTCC
CGTCGAACCGCTCCAGCCGACGGACGGACCCCAGAGAGCACAAGTGAACAAAGCCGCCACCACCACCAGACCAGGGGAAAGGAAAGGGACTGCCGAGTGC

> ZmOSCA1.2

CAACCAGACCCGTCCACAAAGAAGGTTTGGACGATCCATTGCGACGGCGCATGGTGCCATGCGGAGGCAGGCGCCGCTGCAGTCATTACCTCACCAGCCG
GGGTTAAGCACAGATATGCAGCGCGCCTCAGCTTCGCTCTGGAATCCGACAGATGCACAAATAATATAGCAGAATATGAAGCAGTCATCCTCGGCCTCCG
CAAGCTAAGGGCCCTCAGAGTCATCACCTGTATCATCAAGATACAGGTGGTGACTCCAAGATAGTTGCCGGCCAGGTCGAGAAAGACTACGCAGCAAAGG
ACCCCGCACTTATGCAATACCTCTCGGCCATCCGAAGTCTCGAGAGGCAATTCAAGGGGTTCACCTTGCAGCATGTGGATCGGGCCAAGAATGAAGAGGC
CGACACATTGGCCAAGGCTGGCGCCAGGGGCGAAACTCTGCCCTCCGACGTGTTCTTTCATGTCATCGGCACGCCAGCCGTCCGGAGCCCCGAAGGGCTC
CAAATAACCAATGACAGCGAGGGCCACCGCATAGTCAACCTTATCATGACCGAAGACTGGCGGGCACCAATAACCCTGTTCCTACAGGGGTATTATCATC
CAGCCGACATCAGTGAGGCCAAGCGCCTCAGACATTGAAGCCGAGACTTCGCACTGATTGAAGGCCAACTCTACAAAAAAGGGGTCAGTCAGCCAATGCT
TAAGTGCGTCACCGAAGCCGAAGGCATGCAGATCCTACGAGAAGTCCACAGTGGCACGTGCGGCTCACACGCAGGGCCAAGGGCCCTAGCTGCTAAGGTG
ATCCGCCAGGGCTTTTACTGGCCCGCAATGATCTGCGCCGCAAATCGGGTCACACGGTCCTGCGAAGCCTGCAAGAAGTTTTCTCCTCGGTCAGGCAGCC
CCTCGCAGTATACAAAGCTGATCGCCCATACATGGCCCCTCCAGCGCTGGGGCCTGGACATTGTCGGGCCCCTGCCCACCGCTCAGGGGAACCTTAAGTT
CGCCTTCGTCGCTGTCGAGTACTTCACCAAATGGATCGAGGCGAGGGCTGTTTCCACAATAACATCAAAAACTATCCAAAAATTCTTCTGGCAAAACATT
GTTTGCCGCTTCGGAGTACCGTCCGAACTAACAGTTGACAACGGCAAGCAGTTTGACAGCCAAGACTTCAAGGATTTTTGTTTCTCCATTGGCACCAAGC
TTGCCTTCGCCTCAGTCTATCATCCGCAGTCCAACGGGGTCGTGGAACGCGCCAATGGGAAAATCTTCACAGCTGTCAAGAAGATGCTCCTTGATGAAAA
AAGGGCAGATGGACCGATCTGCTACCTGAGGCAGTCTGGGCACTAAACACGACTGAGTGCAGGGCGACTGGGTTTACTCCCTTCCGCCTTCTATACGGAT
CGGAGGCCATGACCCCGCAAGAAATAAAGCATGGGTCCCCGCGAACAGTTCCGTCAGCTGTCCCCGACGTGGACGAGCCAACTTCAAAGGACCTCATTGA
CGGAGACCGAGTCTTCGCCCTACAAGCCCTCAACAAATACCAAGCCCAGACCAAAGCATGGCGCGACCACACAGTCATCCTGAGGGAGTTCAACGAAGGG
GACCTCGTACTCGTCCGAACGGCTCGGACGGAGTCCAAGGGCAAGCTGGAGCCCAAGTGGGAGGGCCCCATTATAGTCAAGACAAAAGCTTCCCCCAGCG
CCTACAGGCTCACAACGCCAAACGGCGAGGACCTGGAGCACTCCTGGAACATCGACAACCTCCGCAAATTTATTGTTTGACTCCTCAGGGCTAATTTCGC
CCTTGTAATTTGCAGAAACAATCTTGTACTGGCCCGCACTCTTTTCCTCCCAGGGGGTGAGGTTTTAACGAGGCGGAGCACGAATCTCTAGAATCC

>ZmOSCA1.3
CCACACCTGGGACCTGGTGCCGTGTCCACCAGGCACCAACGTGGTCACCGGCAAGTGGCTCTTTCGCCACAAGCTTACCTCGGACGGCTCCCTCGACCGC
TACAAGGCCCGTTGGGTCCTTCGGGGCTTCACCCAACGCCCCGGAGTGGACTACGACGAGACCTTCAGCCCCGTCGTCAAGTTTACCACTGTTCGCACCG
TCCTCTCCCTCGCCCTCTCCTAGGACTGGGCGATCCATCAGCTCGACGTCAAGAATGCCTTCCTCCACGGCACTCTGACGGAGACTGTCTACTGCAGCCA
GCCCACCGGCTTCGTCGACGAAGCCCGTCCGGATCTGGTCTGCCGGCTGAACCGCTCTCTCTACGGCCTCAAGCAGGCGCCGCGGGCCTGGTACAGTCGC
TTCGCTTCCTACTTGGCCTCCATCGGCTTCGTCGAGGCCAAGTCGGACACGTCCCTCTTCATCTACCGACGCGGCGACGACACCGTCTTCCTTCTGCTCT
ACGTCGACGACATTGTGCTCACGGCATCCACCGCCGACCTTCTACAGCGCACGATCGTCGCCCTGCAGCGGGAGTTCGCGATGAAGGACCTGGGGCCCCT
CCACCACTTCCTCGGCATAACCGCTGAGCGTCGGCCCCAGGGTCTCTTCCTCCATCAGCGCCAATACGCCATCGACATACTGGAGCGGGCCGGCATGTCC
GACTGCAAGGCCTGCTCCACGCCTGTCGACACTCAGGCGAAGCTCTCAGAGGACGACGGGCCCCTGGTCGCCGATCCGACGTCCTACCGGAGCCTCACCG
GCGCGCTCCAGTACCTCACCTTCTCCAGGCCTGACATCGCCTACGCCGTCCAGCAGGTGTGCCTCCATATGCACGCCCCGCGGGAGCCTCATCTCACCGC
TCTCAAGAGGATCCTACGCTACCTCCGCGGCTCCCTCGACTACGGCCTCCTTCTCCGACCATCCCCGACGTCAGACCTCGTGGTCTACACCGACGCCGAC
TGGGCTGGCTGTCCGGACACGCGTCGATCCACCTCCGGTTACGCCGTGTTCCTGGGCGCTAACCTCGTCTCCTGGGCCGCCAAGCGGCAGCCTGTTGTCT
CTCGCTCCAGTGCTGAGGCCGAGTACCGCGCTGTGGCCAACGGTGTGGCAGAGGCCTCCTGGCTGCGACAGCTCCTCCACGAGCTCCACAGTCCCCTCCA
GCGCGCCACCCTCGTCTACTGCGACAACGTCAGCGCTGTCTACCTCTCCACCAATCCCGTGCAGCATCAGCGCACGAAGCACGTGGAGATCGACCTGCAC
TTTGTCCGGGAGCGTGTCGCCGCCGGTGACGTTCGGGTTCTCAGCGTCCCCACCACGCTGCAGTTCGCCGACATCTTCACCAAGGGGTTACCGTCGAGTG
TATTTTCAGACTTTCGCTCCAGTCTCAACATCTGTACAGGATAGAGTTGTGACTGCGGGGGGTGTTAGAATACCCTCTCACCATCTGTAATGGGCCTGGC
CCAGTTATTGTATTAATAGATCACCCAACCCCTGTTAGGGTTAGGGTTTCCAATAGTTATGTTTGTTGTTCAATTAAGCGTGGAATTGGAAAATTTTGTA
CCATTCATTTTATGTTTTACTTTTGTTATCTATCCAAACATGACCCACTGTGGGTGATTTAGGGGCTTAAATGTTGGCCCTAAGCATGAGAGCTTACTAG
CTCTATGGCCTCTATTATGTATGATAGATAGCTTTTGTCGAGCCTTTTATATATTCTCCCTCCATTTTGGATAATCATCTTCAGAGCATTGATTAAGGGG
ATTAACACTAACTATTGATGCGTTAACAAGAATTTACCAATTCTATGGCCTAGTTTCATTTGATGGACTAACCAGCGGACATATTTATTATTGCAAATGT
TAATGGCCTACTTCTCTTGACTGTTTGTTTGCTTATATGCAGCCTTCTGCTTGAACAGCTCGCTAATAATAATCTATGATGCTTATTCAGGAGTGAATCCCATAATAGAGAT

>ZmOSCA1.4

CCCTCCATAAAAAAGAATCTAGTTGGTGGCTCACAATTATGTCGAGATGGCTACAGACTTTTTTCAGTCTAATAAATGTATACTGTCAAATTATGGAACA
TTTGTTAGAAAAGGCTATGGTAGTGGAGGCTTGTTCCGCTTATCTTTACATGATACGTGGCTCGTTGTAGATCGGACCTTGGTCCGAATATTCCTTTCAA
ACGGTTGCACATTTTGCTTGTCGTGATGACTGTCCGTTTCCTGTCTTCTGGCTGGTAAAGGACGTCAGTTACTCATCGATTTCTAGTTCTAATGGCGCGA
CTGTTTTTGTTCGTTCTCCTTCTCCCCTCCTTCTGACTGCCTATAAGAATGGAGAGCGAGGACTCTCTTCCGGTTACGCACGATCTCTCACACGCGTTGC
AAACAGCACGCCCTCGTCCCATCTTCTGCGAGCACAGAGAGAGAGTGGGACAGCATGACTCCGAAATCGCCGTCCGCAGATAAACAGTTGCACGAGTGTG
TGGGCGATCAGGTTTTTGGGAGCGTATTCGCGACTGTTCGTTTCATTCGTTCGACCAGCGCTGCTTCGTCTACACCCGGTTCTTCGTCGTCTTCCCTGGG
CGACGTGTGTTGTTGTTCTTCTTCGGAAGAACTTTCGAGGGACTGCACTGCGTACATCTTGCTGCATCGAATTTGTACAGCTACATCGAACATACACACG
CGATATCTCGTGTGAATGGAGCCACTGATGCCTTGAACATTGGTCACTCTGCAGGATACTTTCTGTTTTTCGTATTTATGCATGTTTTATTGCTCGTTAC
TATTTATACGAGTAGTGATACACACATGCATATACATATCGTCACACTGGTTTTCTAGATTAAATTAAACCTGATAATGCCCAGTAAATTTCTAACAGAT
TACAACTAAAACAGTTCAAATAGCTAAATTTAATCCGAATGGAACAACCTATCATGTCCTAATAATTATTTTGCAGGGGACGTGCGACCTTGTCTGCGTG
GTGTACAGCCGTACAGGCATTGTAGGTAGTTGGAGGCTTGGAGCACATGACCGGCGATCCCGCATGTTCCGTTTCCTCGGCGGGAGGAGATGCGCTTGAC
GTGTTGACTCTCTGCAAGTGCTCCTCCTGTCAGTGAGCCAACCGTTCCTTGCTTCCACAGACCACAGGGGCTTGGACTTGGAGGTTAGTACAAACCAGTT
GCGGGATTGATTACAGATAACCAGCGTAGGGTAGCTGCCTACCTGTTGCTTGTTACAAGTACTGCCTTCACAGAGAATTTGGACGGACATGTGACACCGA
CGCATGTGGGTCAGTTGGTGGAAAGTTCCAAAAGGTTATCATTCGATTCGAGTGCCATCTCATCCGTGTATATGGAAATTCCAAGCCCGCCGCTTGCTCT
CCTTGTCACGTTGTTCAGCAAGCAGCGCGGTCCTCTGTTTGTCACGTCGTCGCTGGGCTGGCGCTTCCTTGGCTCGCTTCGCTGAAGAAAAGAGAGAGCA
GGGCAGCAGCACCGCGAGGACGACATGCATCCACGACGAGTGAAGACTGAAGAGCCCGTCCATCCACGCTCCACGGGCCGCACAGCGACAGATAGAAACC
GGCTGTGACCAACTGGCCAGCACCACGCAACAGCTAGACTAGACTGGTAGAAGAGCTAGACTGCTAGAGCCTAGAGCCGCCGCGTCCCGCGTGCGTCGTG
CGGCGCTCTCCCGATCCCGAGTGCATGCTACCTCACCTGCGTGTGCGCCGCGTTTCAACTCGAGAAAGAAGACACACCGTCGCGGCGCACGCGGCCTCTG
GTTTCTGATTTCCTGGTTACCCTTTCCCTCCCTGCATTTGCCTTTCAGATCAGGCGGCAGCACCTTCCTCTCCTCCCCTTCCCTTCCGTCTCATCTTCGT
TCCCTTCCGGGGTCATCCCTTCCGTCCTCCTCCTACCAAGTCCCAAGGCCGCGAGACAGACAGACAGACAGACAGAAAGAGAGAGCAGAGCAGACA

>ZmOSCA2.1

GGTACCTTCGGATGCCCGAGGGGCTCAAGTGTCACACCCGGATTTAA
GGGACAAAGCCGCGTGCGTCTTATATGTGCGCCAAGAAAGAACAACACATATAATAACAGAGTGCATAAAGATAGTGTCAACACCCGGATTTAAGGGACA
AAGCCGGGTGCGTCTTATATGTGCGGCAAGAAAGAACAACACATATAATAACAGAGTGCATAAAGATAGTGTCAACACCCGGATTTAAGGGATAAAGCCG
GGTGATCTGGAAAATACCCGGAATTAGGAACAAGAATTTTGTGGCTTTTGCTTTTTTGTGTCCATTGTTGAGGACACCAGTAAACAAAGTGTATGTGCTA
TTATTGAATAATTTTGTTGTTTACATAAACAATAGCATGAACTCGTGACTCCGTTTGTGATGCTTGATAAGATGGTGTCTACAATAGTTTAATTTGGTTT
GTATCAGATTAATATTATTGGTTGTTTGGGTGATTGATGGTAATATGTGTTGCGTCTTTTTATGTATATGTTTTACTAATATTTTATCGAATAAATTTGT
GTGTCGTCCTGTTGGTTGTTATACATGATGTAGGTGTTTTCAATACATGTATGTTTATATGTAAAATGTGTGCAACTAATATATATTAAAATTGAATAAT
TTGTGTGCCGTCGAGGCATTGTACTAGTAAATTATAATATCTGTCATTTAAAGGCTGCCAAAAAAACGAACGATTGTATGGCGATAAACCTTGTATACGT
GACACATATGGCAGGTATTGATCTAAAACAGGCGATACACACATTACGATTGTATAGCGATAAACCTTATATACGTGACACATATGGCAGGTATTGATCT
AAAACAGCCGATACACAGATTTTGTTACTCTAACAATGTAAAATGGAAACAGATAAATAATCCTACGTGTTGATACGGGCCGGGCGAGGCCTGCCTTTTC
GACTGTGTGAAGGCACGGGAGTTAAATATCGTTGGTAACAAATGGTGGGATTTGATTTTGTGGAGAAACGAGTGGATGGACGGAATTGGACAGCAGTCGA
TGAAGAAGCAAGGAAGGAAGGGTCGCTGTCTAGTTTGTCGTGGCCGTAATTCTTGTGGTGGTTTGCCTGCTACTACTGCTTTTTTTCCGTGAGCTGAGCT
GAGCTGGTCCTCGTCGTGCCGTCATCGTTCTCCAATCTTCCATGCCTACGCGTCGACAGGTCGGCGGACCGCGGCCGCTGCTTGTGGGCGCCGTCGCGCC
AAACCGCGCCCACCACGTGCTGCCTACTCACAACTTTCACGGTGCCAAAATGATGGATATATGTGTGCAGCACGGCTCGCCGGAGCCTGGCCAAACACAT
ACGTTCACACGTATCCACATATAGGTAAAAGCCTCTGAAAAAAACGTAAACTACACCACGCATACCACCTGATGGCATCAATCAAACATATACAGACACT
TAGTCAACATATACGATCAGCCAGGCTACCCACAACCAAACACACGCAAACTGATTATTACCAAGAGATTTGAACAGGCACCATAAGGAATTCGTTTGCT
TTTACTTAATTATATGCTTTAATGAATCCACACCTTTATTTGCTATATAATTAGTTTATTACCAAATTGTTGCAGTAGAAACAAGATTACATGTATAAAA
AAAAACAGACTCCCTTCGCTGTCTCGTCTTCCCTGGGCTCTCTCTCTCCCTGCAAAAGGGCCAAGGCAAGGATTCTCGTTCTCCTCCAGGTTGGTTTGGA
TTCCTGTTCTGTTCGCACCGCCTCCGCCTCCCTCCCTCCCTCTCCCCTTCCGGACTCTCTGCTCTCCTCTGCTGCTAGTCCATCCATTAATCTCCTCTCC
TCGCCATCGCCGCACTCACGCTACGCTTCCCTCCTCAAGCCCGAGGTACAGTATCTATTCCCGCTCTCTCTTTTCTTCTTCTTTCTCATAATACCAAACC
AAACCATGCTCTGCTCTACTAG

>ZmOSCA2.2
TAAATTTACTTGTCAGAGCAAGGTTTGAAACACCGACACTACAAAAATGAGATATCTCCCTACGGACATACTCATCTTCTAGCTCTAGACTTCTCTATAA
CCAAGAACACCGTGCAATACAACACTACAAACATACATGATGTAAGGTATTACACGCTTGAGCAACCCGAATCTGTAAAAAAATCTTCTATGTTCCATCG
TGCCCTGCATGAACCATCGAGCCACAATCGACTTTGCCATCCTCACAAAAACACTACATGGTACTTCCCGTGCGTACCATCGGTATCAAACTCCAACACA
ACACGTGCTGAGCCATGCCAAATAAAATAATGGCGCATACACCTTCTGTACTATACCGCGTGCAACCATGCATGCACACAGAGGAGTAACAATATGGACA
TATACACTGAGTGTGTGGACAGAAGCATCGATTTGGCTGTGGAGAAAAGTGGAAAAAGGGACGAGAACATCTGCAAGCCTATGTCGGTTTCCTTTGTTTA
AGACTAGTTTGACAAGTCCATTTCCCTAAGGATTCTCATCTTTACAAGAAAAAATAAACTAATTTTCCTTAGTAAATGATTTTTTTTGGAAAAATAAGGT
TCCTTAGTACTGGTCTTGATCCATTTGCTTGTCCATAAGCCAGCTAGTCTACTCTGTCATCTGCAGACAAACTTTAAATCTTTTGACATTTCTAGAACAA
AATTTAAATTGTATAGTTTTTAAAAATATATATTAAAAAACCAAAACATCTTATAGTTTAAAATAGAGGAAATACATAAATAGTCATATAAAAAAGAAAC
AACTGCGTGAGTCAACAAAATCTTTTCCAAACTTATCGCTTTACCTTTTGACACTTATATAATTCCTTATTTTATTATAGAAAAAGATATTCCATCCATC
CATCAAAATTATATATATGCCGTAACTTTGTTTTCCTAGTCCGGCCTTATAATTTGAAATTAATTTTAAAAGTATATACTATAATATTTTGTCATCAAAT
TAATCTTATTGAATCTCATAAGTATAATTTGATGGTGAATTTTTTATGTTATTTTATAAATGAATATGGTAAATTTGATTCAGGATAAAACTACTAAATT
GATATGTACTTTCTCTGTCTCAATATATAAGACGTCTCATTATATAAGGCGCAACTATCTCTATTCGAAGACCAAGAAATATATTTAATTATCTCTATAT
TACTGTATCGATATACGCTAGAACATGTCTTATATTATGGGACAAATAAAAAATGGTTACGTCTTATGAACTGTTACAGAGAGATTCTTCAATTTAAGTC
GGGCGTTCGATATACAATGCTACATGACTTGGATGGCATTAAGAAAATTTAGTTAGAAAAAAAGCGCGATTGACAATGCTACATGACTAGGAGGACATTA
AGAAAAATTTTAATCATGGCTTGCAACTCCTACAAAGTACGTCTAAGCCCACAGCACAGGATCTCTTGGCAATTGGCATCCATCCGACCATCCCCGTGGC
ATTGGGCGGTTGGGTCCCGCCTCCCTCCCTCCACCTTTTCCCCGTGGGAGCATTTTGACTCACAGGCTCGCTCCGCTCTGCTCCGTTACGCCGTTCCAAC
CGTCACTTTCCTTTCTGTGACCCGCAGCCGCAGGCCCATCCTCCATGCCATCCTTTCCTTCCAATCGAAACTTCCAAATCCGATCCCACCCACCGATCGC
CAACTACTCCTACTGCTATAACCATCCTCCCCAGCGCCCCGCCCGCCCTCCGTGCCCCTCTCCACTTCGATCGCCTTCCTCGACAGTTCATTGCCATCGG
CAGCGCGTTGCCGCCGCTCGACTCGACTTGACTCCACTCTGCTCGAGGTGATCACCATGGTTGCTCTGTTCTGTATATGATTCTAGCCGCAGAGTTTTTT
TTTTTTGCGGGAGTTTTTTCCCTGACTTTTTTGGGGTCATCGATGGATGTGTAG

>ZmOSCA2.3

CGCGCCTACAGGCTGGACGGTCCACGCTAGGGATGGCAACGGGGAATTCCCCACCGGGGAATAGCTCCCCATCCCCGTCCCCGCGACGAAAAAACTTCCC
CGCGGGGATCCCCACGAACGCTTGCGGGGAGCATTTCTTCCCCATCCCCGTTCCCCGCGGGGATAAATCCCCAACGGGGATCCCCATCCCCGTTTAAATT
ACAATTAAGACATACATACTTTGCTATTAATGTTAAATATTGTCGCTTATACACATTGTCAGATATAAGAAATCATTATGCACACATCAAAATAAACACA
TTTGTACAATTAGTCGTCTCTTGATAAGGTAATTATTTATTTTTGTCGTTAACTAGTACAAAAAACATAGTCACGTGCAAGTTTAACGGGTCCCCGCGGG
GAACGGGGATGGGGAATGCTTCCCCGTCCGCATCCCCGTTTACCCGTTGGGGGATAAAATTTCCCCGTTTATATCCCCGCGGGGGAAGTTTCTTCCCCAT
CCCCATCCCCTAATGGAGGAATTCCCCGCGGGGAATCGGGGATCGGGTCCCCATTGCCATCTCTAGTCCACGCGTGCGCTGGGGTGGCGAAGGTCACTAG
CGGTGCCTGGATCTCGCTCCCGGGAGGGACCTCGTCGGGGAGGAGAGATCCTATGGGTTGTCTAGGCTCGGCAGGCCGACCTAGACTACTATAATCGACG
TAGAGTTGAAGAGAAACAAAGAATCTAGGGATTGGAATACTAAACTAGGGCTAAACTAGAACTACTCCTAATGCGTAAGGTAAAAACGAGAAATAGATTT
TATTTGATCGATTGTTGTGAGGTTCAATCGGCCGTAGCCCTTCATCTATATAAAGAGGATGTCTGGATCCGCTTTGAACTAATTTCCAAATCAATCCCAC
GGTTTTAGGTAACAAATCCCGCGAGAAACTAGAAACTCTAACTGACTCTGCGTATGCATGGACCGTCCGGTTCAGGACCGAACTATCCGTGAGGCACTTT
TGGTCTCCAACACGTGGCAAGGAGCAAGTAGATTGAAGGCTGTCGTCTATCGATGGGATAGGTGTAGTGGCTTTGGAGATCGATGGTCATTGTTGTGTCA
ATCATGCTGGGTTTGTGTGCGGGAAGGGGTTGGGCGGACCGGTGGAGGAAGGGGCTACAGGGGACTAGGGGAGTTGGAGAGGGAGCTCGGAGCCTTGGGG
CACACAAATCATGCGCAGACTAGGCCAGGTCGGCCCACCGTGTTTCAACCCAAATAGGTTTGGGCTGAGAAAGCCCAAAAAAAATATGAATCGTGTTGTG
ACAATATAAAAAGTAAAATTAGCGGTCCAACCTAGCTTATCGGGTCATGTCAGACCTTTTATTTGGTCGTATCGGTCAAACCTGACCTATATATTTAAAC
CATATAAAATATAAATATTATAACCATATATAGCTTTCTAAATTAAAGAACTAAACACCGGGCATTATCGGGTCGACACGGTCCAAGTCCGGCCCATTTG
GGACTGTGTCGGAGGTCCGATAGACCAGAATTTGAGGTCCAACACACGACTGCCTTTGAGTTGTGCTAGTTTAGTATATTCATTTCGTGCTAATCTTTGT
TTTGAGCTTTTTTTTGGACCGTGCTAGTGTTGGTGCTGGTCATAAAAGTCGGCCCATATTTCGAGCGATGACGCATGGTTGTCCGTGAGAGGGCACATAT
TTCTGTTTTATTGTTGCTGCTAGATAGACTTGGGTATTATTATTATTATTATTATTATTATTATTATTATTATTATTATTATTATTAAAGTTGTAGATAT
AGAGGTAGTCAGTTTGAAAACTAAAGTTTTTTATATTTTAAAAGGGTTTTACCAAGCAATGTTTCTCCTTGACTTTTATGGATGTGGTCGGAGAGATGCT
TCATGCCGACCAGTCGAACCCTATAGAGCCCCACGCCATGCCATCCTCTCATCTCAAAAAGGAAGAAAATAAAAAGAAAGAGGACAGAAAAAATCGTAGC

>ZmOSCA2.4

TACCTTTATCATTTTGATCTATAGAGGTATGTAATAGGCCAATTTTCAAGCCCAAAAGTGTTTTTCTTATTTTCCTCGATTTGCTTTCCAAGCACCCATG

CTCAGGCTCATATGAGAAGTGCTAGTTCATAGTGTTGTGTTGAACAATTAATCACCAAAGCACTTAGAAATGCCCTAAGTGCACATTTCCCTTTCAGAGG

GTGCATCAAGACAAAGCAACTCCGTGAAGTAAGTATGGTCATCAAATCATCGTATTAGAAGTTGGTCTATTGTATCCCTAATTGTGTGGATAAACTCTAT

GTATCAAGATGTAGTTTATGAATGTGAAAAAACTCTATAAATAAGGAGGAAGAATTGCACCAACTAGTTATCTCTCTAGCTTTTGGTTTTTGCTCTCTAC

TTTTGTTAGGAGATTCATAGGTGCTCTTATTCTTGACACTTATCTTCTTAATTATGAATTGGGAAGTATGGCCTTAACCACACCGTGACCTTAGTGGTAT

ATCTCTGAGCTATCTCTATATGTATCTTTTGATGGCTTGAAGCTCTTCCCAAGCTCTTCATTTTGATCTTTAATGAGATTTTATAAATATAAGTTCTTGG

GATCATTTGGGATGTATAGAAACATTCTAGGACCTCTTAGGATCATTTCCACCAATGGTATAGACCGGTCCACCTAGAGCATGAATTTCTAATCCTTTGT

GTCATTTTGAGAAAAAAAGAGTTGATTCAAATTCATATTTTTATCTGACTTTGAAATTTGAATATTTTTAGCTCAATTTTTGTGGAAATCTGGGACAACC

TTATGAAGGTGTGTGCATAATTTTAGCTCAATCGGACTTAGTTTGCATATATTTTGCATTTTAGAACCTTGATTCGGGTTGTTGAAAACTGGCCAAGCAC

TACCCCTTGAAGAGCAGTAGTGTCGGGCGATGCCTAGAAGTGCCATGGTGTAGCAACACCACTTGTGGAGTAATATTGTCGAGCCAATCTGGGTGGGACA

CTTTTTTTAGGGTCGCTTCAGACTTCTTGAAGTTCGTTATGTGATCTGTTTGTTTCATTGGACTCATGTATGCATTAGTATTTTTTTCGAAATATATTTT

TGGTTTGAGCATTTTCTTTATCCCAATTGAAGATAAAGAGCTCGATTTCCTGATTTGAGATAGTTTTATCCGCAAACTCATTGATGACTGTTTTAAGAGT

TTCACTATGTTTTTCGGTCATCGTCGAGAATATCCATCTACTAGTTGATGCTTAAATACCTCTTTTATGCCTTTTGGAGTTTTGAACGGTTAGAGGTAGC

ATTGGAAAATAAGCCAACATTACTTGAGACCCTCTGTACGGTCGCCTGATTCGATCTTCATATATAATCAGAAGAGTTAAATTTTGATGAAATATATAAT

AAATTTTATCATCATTTGTGTCTCTAATAAAATTTACTACAAAATTATAACTTATTATTAATCTAATAACACTTATTTTATATCATAGATACTGACATTC

TTTGTATAATTTTAAGTTCAAATATTGTAAAAAGGAACATCCCATTTTGGATTGTCTAATCTTTAAATATTGTAAACAAGCAATCTCCATTTTCGATTAC

AAATATCGTAACGAGACTTTGTACTACATACAATAAAACAAGAAATTATCCATAAAATATTGTCATCGCATGATTTATATTTAATGACACGTATTGGTTG

ATTGCACAAAACATATATTTGCACGTAGGAGTACCTCCCATATATATACACACGTTACACACGTCACGTAAAACGGGTCCCACAATCGCACACGTCTCGC

TTCGCGATAAACCTGTGCCGACCACCGACCACCACGTCACAGGCTCACAGTCACAACCCGCCTGCCACGTGCTTAACTGACATCTGGGACCCACCGTACC

CACCACAACAATTTTCCATCCCAACCGCGATCTCGTTCTTGTCTTTCTCACTCACCGTGCGCGGCGGCAGCTTTGCTTCTTCCACCACTGACCAGCGGGT

CCCGCCTCGCCGGTGGAGTTTTCAGGGAGCTTCTTCTGCCCGCCGGCGCG

>ZmOSCA2.5

TGACGCCAGCTCTTCACTCACTTTAATATCCGAGCATTCGCGCAACAGCAACACAAGGTCCTTTGATAGGCTTTTCATCCTATTTGATTCTGTAGCTCCA
TCCGAGGTTGATGTTACGTGTGATCCTGATCCATCGGAAGGACGGTTGCTCTGGCAGATACTCTCGTGGAACATCTGGACAGCATCATCTGCTGATATTT
CTTGCACTGAGCGGTGCAGGTCCTGTAATTTCCCCCTACGCTGAACATCCGGCACCAGTTTTTCTTTCAAGCTGTCAGGAAGACTAGCTAGCGCACTGCT
TGAAAAGATGACAGATGGAAAATTAAAATCCAAGGAAACACTAGAATAGAATGATAATACAAGACATCTATTATTGGAAGGATAGAGGAAATGCACCTGC
CAACCCCACGAACAAGGCCCCATTTGTAGCCAACGTTTACAGATTGCCTGAACCCAACATTGAAGCCTTCCTGGGCAGAGTCCTTCTGCCCTTCTGTTAC
ACCATCACGATATCCCATCTAGAGTGAAATCAAAAGCAAAAATATTTTAAACATTTCTTCTATCAGTAAAAATAACTGCAGAAGAGTAGTTTGTGCTAGC
AACTGTGTGCGTTCCAGATAAACTCTAGGATAATTAATACCTTCTGAAACTGACTCTGCCTGTGGATCCATTCTCTGTCTAAGGTATCCGCGTGCCGAGG
AGAACCTGAAACATCCTCCCATACATCATCGAGTTCGGCAGCAGCAACATCGCCGCCATTAGCTGAGACAAAGTTTTGCATAATCAACTAACTGCAACAT
GAGATAAGATAGCACTAGTGCAAGCAGTACTACTAATTAAGCACAAGACATGGTACGGAATAACTCCCCTATGAAAAAGATCAAACAGCGCCCGCGGTTG
CCCGCTTGTGAGGGATACGCACCCTGCTTGGGGACTTGAACCTCAATCGATGAAGGCGCTGCTTCTACGCTCAGCTCCGACATGGAGGAGCTGACGTCCA
CCGCATCCCTGGGATTGGCCATTCAGAAACTCCAACCTGCAGGAAGCAGCCAAAGCACCGCCTGTTGCGAGATGGAAATGTATTTGGGGTGCTCAAATTT
TTTTCACAACCAAAAGGCTAATATTTAACATGTACAACGCTGCATAGTCAAAATTGCGTAATATATGTAGGAAAAATATAACCCAAAATTGATCATGATT
TCATATATTGAAGAATTTGACCATAATTTGAAATTGTTTAACATAAAAAGATATCAATGGTTGAGACCTAAAATTAAAACTGTTTGACACAAAAGGTTAC
CAATAACTGAGACTTAAAACTATATCAGGTGACTAAAGTAATTAAGGATAACTCAAGATAAGGCGCTAAACGATTCAAAAATTGCTATCCCTGTTCCCTA
ACAAATTGAATCTGAATTTGGAATAGACTAGCCAGACATCAGTCACGCGCTCACCACAGCGGAGATGCAGCGGGGCTGGATCCAGAGGCTGTATCTGCCT
CCGCGGCGGCGCAGGAGGAGCGAGGAGCCGAGGAGCAGGCCGGAGAGATCTGGAGTCGCCAGGCGGCTTTCCGTTCGCCGCCTTCGCTCGAGGCTTGGTT
CGCCGCCGCGGTACTCCAGTCGCGCGACTCTGGCCCGCGGACGGGCTGAACGGACAGATTGGGCTAGATTGAGGCCTGTTGGGCCTTCGGACCTCGCCAG
AATCCGCTACAAGGGGAGGGCCCGTTAAAGCACACCCTGTGGCCCTTGGCAGGTGCGCCAAACTGTGGCAATGCCCATGCGGTTGCAGGAATGGGTGACT
GTTGCGGCGGCGCCAACCAAACGCGTCGTGCTCGCGGCAGGCCACGTGTCCCTCGAGGCTCCTCCGGCGACCACGTCGCCGCCCGCCATATGACCAACGC
ACCAATCAGACTCCGCCGGCGGGAAGCTGATCGTTCACTGCTACTAGCGCAGTGCCCACGCTGGTGCAGCACCGGATCATCTGTGTGACAAGGAAGAAGA

>ZmOSCA3.1
TCCTTTCTCAGTCATGACACTTCAAATTTGTGCTAACATGTGCTCTATCAATAGCCAGGGACCAAGTAAGCTACAATTTCTTTTGGTACACATGTACATG
TACTTATTTTCTATTTCATGTGAGGTCAACATTGATATGAAGGATTATGTATTAATGTATGCTTTACATGTCTAAATAAGGCTTAATCATTAGTTCTGAT
TTTATTACCTGCAGGGTGGTGAAGATGATAACTTTGCTATTGTGTTTGCTGCTATGGGAGTGAACATGGAAAAAAAACTCAATTCTTGTAACGTGGTTTT
GAAGAGAACAATTCCATGGAACGGGACACCCTTTTTCTAAATCCGGTACCTACAAGGACTTGTGAATTTGTGATCAGCATTCACTACCATTTTATTTCTC
CCAAGAGTGTCATAGCGAATTTTGTATTGTTTAGATACATCTTTACCAAAAAAAATCCCACATATTTAGTGTCGGAGCTTGAACAATAGCTTAATGGAGC
TATCAATGAATTAGGGGGCACTAATGATGCATGAATCAAATTATGTTTGTTGAGAAAAAACTAAGATCCTAGGGCAGTCATGGCCCAGGTTGGCCTAAAC
TATGCTCTAGCCCTACATAATGATGAGCTAAACATATTCTAGATCAGACATAATCAGTGTATTGCACAACGTACTTATGCAATTTTAGCCTTTGCCCTTC
TCTTTCAGGTTCTAAATAAGTCTTGCATGATGAAAGTATGTGTTGTACGCATAGAAAGTGTCATGACAGATGTTCTTCGCCTCTTCGAGAGGTGATGATT
GTGTCAGCTTATCTGTCCATGTAATGCTCATACTTTCTTTTTGTCATGCATATGTGACTTGTAGACCAAATTTTGCCTCTAATGAGCGATATCACTTATA
GACCCTATTCTTTTATGCATGCAATATCATATGCTATCCTTGTAGTTGTGGTTTGAAACAAGAATTATCAGATAGCTAATAATTTCTGACACGACCTTGT
AAGGAGGCCTTTTGGGGGTCAAATATACAAATGTAGTTATCGTTTGGAGAAGTAACCTTGTGAAGCATTTTCTTCCAAAATATTGATGCTGCTTGTTTTG
CACTCATTAGATTTAAGCGACCATAATGTAGGACCACACAAGATTCAAGGAATTGGAGCTGGTTTAATCCCTAGAGTCTTGGATGTTGACCTCCTTGATG
AAACTCTACAGGTGTGTTCTCCTGTATTAAGTCGGGTTGTAGAAAGGACAGACTACCTCTGGTCCCAAGTATAAGTCCAATTAGGATTGTTGGTTACAAT
TATTTAGCTTTGACTATTGATTTCTACGAACCATATGAACTATTTGGACTGGCATCATAAGAATGTAATTACTAGCTTTATCCTAAAAAATATTTGTCTA
ATAACTTTGTTTTTTATTTGATTGAAATAATATATGTTCATAGTACTATTAATCAAAGTGGTGCACTGCAGATCATACCGGTATCGTATCAGACTTATAT
TTGGGATCGTAGGAAGTATATAAACAAGCATGCTATTTTTTCACTCTTTGGTTACAAGTACTGTTTTTCCCTCATTATTGGATGTTTTTGGTTGATCCAC
ATTTATCTTGTACTCACATGTCTTTGCATCCGCTTTCTTTAGCTGGCATAACCTTTTGTCACCTTCAATTGTTTTCCAGGTTTTAAGTGGTGAAGCTATT
GATACTGCCAAGGCTCTTGCTTTGAAAGAATCGAAATAACGTACATAAACAATCTTTGAATCTTGTGGTTGTATCATTTCTCCTCATGTCAATGAATGTA
CAACGTCCTTAGGTGCTTACAATATTGTCACTCAGTCATATTTGTGCTACTATTCCATCATTTTCCCTTGCTTATCAATGAGAGCCTTTATGTGAAACTA
ATGCTTCATGAATTGCTTTCTTTTTCACAATTGAATGTTTTCCTGGTAGGATTGATGTTCAATTATTTCATAGGTGGTAAAATGTTTTGAACCCCGAGCT
TGTTAAAGGCC

>ZmOSCA4.1
CCAACGACTATTTTGGTGGTT
GGGGTTATAAATACCCCCAACCCACCCCACCATTCATTGCATCCAAGTTTTCAGCCTTCAAACCTCATACAAGAGCTATAACATTCAATACAAGACACAA
ACAAGAGATCAAATCCTCTCCCAAGTCCAAAGATCATTCCAATCAAATAGTGACTAGTGAGAGAGACAATTGTGTTCCTTTGAGCTCTTGCGCTTGGATT
GCTTTTCTTCTTCATTCTTTCTTGTTTCCAACTCAATTGTAATCAAGGCAAGAGACACCAATTGTGTGGTGGTCCTTGTAGGGACTTAGTGTCCCGATTG
ATTGAGAAGAAAAGCTCACTCGGTCTAAGTGACCGTTTGAGAGAGGGAAATGGTTGAAAGAGACTAGGTCTTTGTGACCACCTCAATGTGGAGTAGGTTT
GTAAGAACCGAACCTCGATAAAACAATTCACCGTGTCATCCGTTCTTATTCTCTTGTGATTTGTTTTGCCATATCTTTCGGACTCAAATTAAATTCTAAC
GCTAACCCCGGTTTGTAGTGTGTGCTTAATTTTATAAATGTCAGATTTGCCTATTCACCCCCTCTAGGCGACTTTCAAGCTTCGTTTCCTATCGCGAAGA
TTCTCGTGCCATATCAAGCGTAGAATTGAACGTTGAGCGATGGAAGAACGCAAACCCTTGGGCCTTTGACTCACCCAATTCCAACCGGCGGAACCTTAGC
AACTAGGTATTATGACGACGATGAGGGGCAAATCACCTGTGCAGACGTCGCCATGAGAGAATGGTAAGGCGACAACAATGACAGTATGTGCGGCTCCGGT
GGCTCCCACAGGATCCCGGAAGTGAGAGCCGAGTCGTCGAGAGAGGGAGCTTCGTTTCCTATCACGAAGATTCATGTGCCATATTAAGCACAGAACTGAA
CGGTGAGCGTTGGAAGAACGCAAACCCTTGGGCCTTTGACTCACCCAATTCCAATCGACGGAACCTTAGCAACTAGGGATTATGATGAAGATGAGGGGCA
AATTACCTGTGCAGGCTTCGCCACGAGAGAACGGTAAGGCGACAACAACGATAATATGTGCGGCTCCGGTGGCTCCCACGGGATACCGGAAGTGAGAGCC
AAGTCGAGAGAGGGAGCTTCATTTTCTATGGCGAAGATTCCTGTGCCATATCAAGCGCAGAACTGAACGCTACAGCGGCTTTAGCCGTTCCCGACCGTTG
TGAGGTGATCGAGCGGCCGGAAATAATCGCGATAACATGGACACTCTTAGGGTTGATTTGGTGACAAGGGGATCACGGGAGGATTAAAGGGGATTGAGGG
AGAAATAAACTAATTTCCCCCTCAATCCCCTCCAATCCTACCGGGATCCCGGTTCACCAAATCAGCCCTTAGAGGCTAGCTTTGCTTCTCGGTTTAATAT
ATAGAAAAATATATAGAAATAGAAATATAGACATGGTGGTAATTAGCAAATATAAGTTATTTCATTCACCAGTACGGCACCGCAGATGCTCGAAAGAAAT
ACCTCTTCTCCACTCCACCTCCGCGTCCCCACCTTCAGCGTCACGCACCTCGACGCGCAGCGCATGGCGACTCGGCTCTCGTCCACGGGCCCCTGATCTA
GGCAGCCGGACCCCGATCGGTTGCCCGTGCAGCGCGACCCCCACGACCAGGATCCAGGGAGTCGGGCCACGGTCCTCGGCAAGGCATCTCATCGGCGAAC
GTTTTTTCGAGGCCGGGCTCCTGCTCCCCCGGAATCTAGGGTTCGCCTCGCCCCGCGTCGCCCTCG
